# Dendritic cell hybrid nanovaccine for mild heat inspired cancer immunotherapy

**DOI:** 10.1186/s12951-023-02106-8

**Published:** 2023-09-26

**Authors:** Chen Shi, Chen Jian, Lulu Wang, Chen Gao, Ting Yang, Zhiwen Fu, Tingting Wu

**Affiliations:** 1 Department of Pharmacy, Union Hospital, Tongji Medical College, Huazhong University of Science and Technology, Wuhan, 430022 China; 2 Hubei Province Clinical Research Center for Precision Medicine for Critical Illness, Wuhan, 430022 China; 3 Affiliated Hospital of Yunnan University, Kunming, 650000 China

**Keywords:** Dendritic cell, Mild heat, Cell membrane, Immunotherapy, Nanovaccine

## Abstract

Cancer therapeutic vaccine can induce antigen-specific immune response, which has shown great potential in cancer immunotherapy. As the key factor of vaccine, antigen plays a central role in eliciting antitumor immunity. However, the insufficient antigen delivery and low efficiency of antigen presentation by dendritic cells (DCs) have greatly restricted the therapeutic efficiency of vaccine. Here we developed a kind of DC hybrid zinc phosphate nanoparticles to co-deliver antigenic peptide and photosensitive melanin. Owing to the chelating ability of Zn^2+^, the nanoparticles can co-encapsulate antigenic peptide and melanin with high efficiency. The nanovaccine showed good physiological stability with the hydration particle size was approximately 30 nm, and zeta potential was around − 10 mV. The nanovaccine showed homologous targeting effect to DCs in vivo and in vitro, efficiently delivering antigen to DCs. Meanwhile, the nanovaccine could effectively reflux to the tumor-draining lymph nodes. When combined with near-infrared irradiation, the nanovaccine induced effective mild heat in vitro and in vivo to promote antigen presentation. After administrating to MC38 tumor-bearing mice, the hybrid nanovaccine effectively promoted the maturation of DCs, the expansion of cytotoxic T lymphocytes and helper T cells, and the secretion of immunostimulatory cytokines, thereby significantly inhibiting tumor growth.

**Graphical Abstract:**

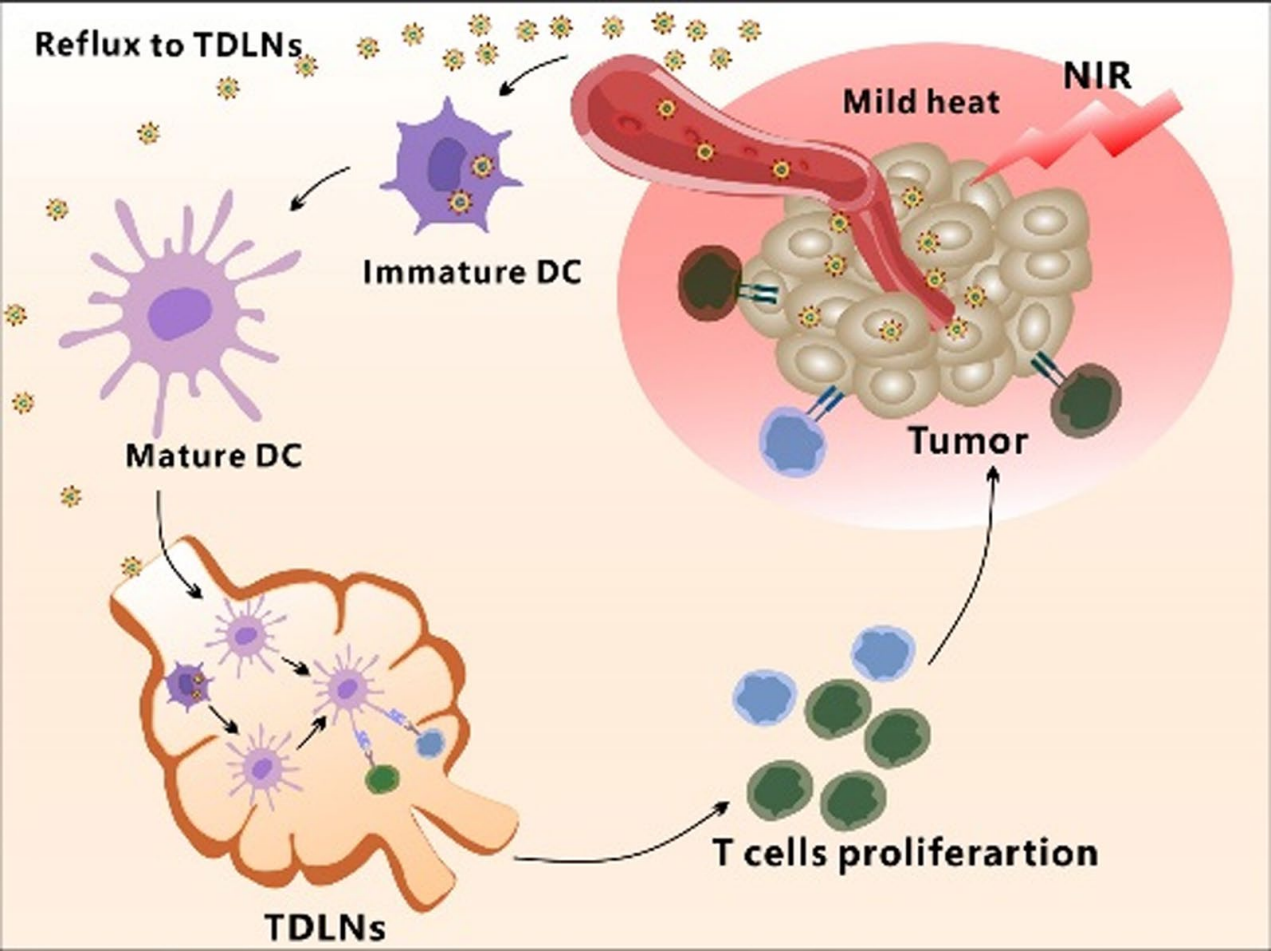

**Supplementary Information:**

The online version contains supplementary material available at 10.1186/s12951-023-02106-8.

## Introduction

Cancer immunotherapy activates the immune system to selectively kill tumor cells, which has been proven effective in the clinical treatment of various tumor types [1]. As the most potent antigen-presenting cells, dendritic cells (DCs) can initiate, regulate, and maintain specific antitumor immune responses, thereby playing a crucial role in cancer immunotherapy [2]. In 2010, the U.S. Food and Drug Administration approved Provenge, the first dendritic cell vaccine, to treat prostate cancer. However, the production of this DC vaccine was quite complex and expensive, costing up to $93,000 for a treatment cycle [3]. In addition, DCs are prone to inactivation or even death during the transplantation process, resulting in fewer active cells reaching the lymphoid organs and thus diminished clinical outcomes [4]. Compared with DC vaccines produced in vitro, subunit vaccines can deliver tumor-associated antigens to in vivo DCs to achieve DC maturation in situ, inducing durable and efficient T cell responses [5].

With the development of vaccine preparation technology, it is possible to synthesize tumor-specific antigen peptides by biological or chemical means. Tumor associated antigen peptide-based vaccines have been widely used for cancer treatment, with the advantages of high purity, low toxicity or infectious factors, and being presented directly by DCs without intracellular processing [6–8]. When administered in vivo, peptide vaccines need to be effectively captured by DC, induce sufficient DC maturation and migration to lymphoid organs for antigen presentation. However, in the physiological environment, antigen peptides are easily degraded by the proteases while being difficult to recognize by DCs, resulting in their short half-life and insufficient antigen capture [9]. In addition, DCs resident in subcutaneous tissues is relatively few, the direct antigen stimulation by local antigen capture may be not enough to cause effective DC activation [10]. Meanwhile, the antigen-primed DCs show poor reflux to lymphoid organs, with less than 5% of mature DC migrating to tumor-draining lymph nodes (TDLNs) [11]. Therefore, it is critical to promote antigen capture and presentation by DCs, as well as TDLNs draining to improve the therapeutic efficiency of cancer vaccine. In the past decade, nano-sized carriers have been prosperously developed, which can effectively load therapeutics including antigenic peptides, protect them from degradation in vivo, and extend the half-life of peptides [12]. Among the nanosystems, inorganic nanoparticles own controllable particle size, regular morphology and surface modifiability, and have been widely used for vaccine delivery [13, 14]. Functionalizing the nanoparticles with cell membrane or membrane components is a commonly used strategy to endow the nanosystem with high biocompatibility and biomimetic properties [15]. Herein, it is possible to introduce the ligands and molecules of DCs to the surface of nanoparticles. Due to the existence of these specific biomolecules, the biomimetic nanoparticles could acquire a high affinity to homologous DCs, which would facilitate antigen capture and presentation [16].

Photothermal therapy is a local treatment that utilizes internal heat generated by a near-infrared (NIR) laser [17]. As a typical paradigm for precision therapy, photothermal therapy is non-invasive with controllable irradiation and temperature [18]. However, the hyperthermia generated by irradiation would kill tumor and immune cells indiscriminately, which would damage the immune system in tumor microenvironment. Recently, it was found that mild photothermal therapy (approximately 42 ℃) not only showed good biological safety for immune cells but also had a significant effect on immune stimulation [19, 20]. On the one hand, mild heat can produce danger signals and inflammatory factors to promote the maturation of DCs [21]. On the other hand, mild heat can promote the migration of DC to lymph nodes for antigen presentation, and infiltration of immune cells to tumor microenvironment [19]. Herein, combining mild photothermal therapy with peptide vaccine could be a promising immunotherapeutic modality in cancer treatment.

**Scheme 1:**
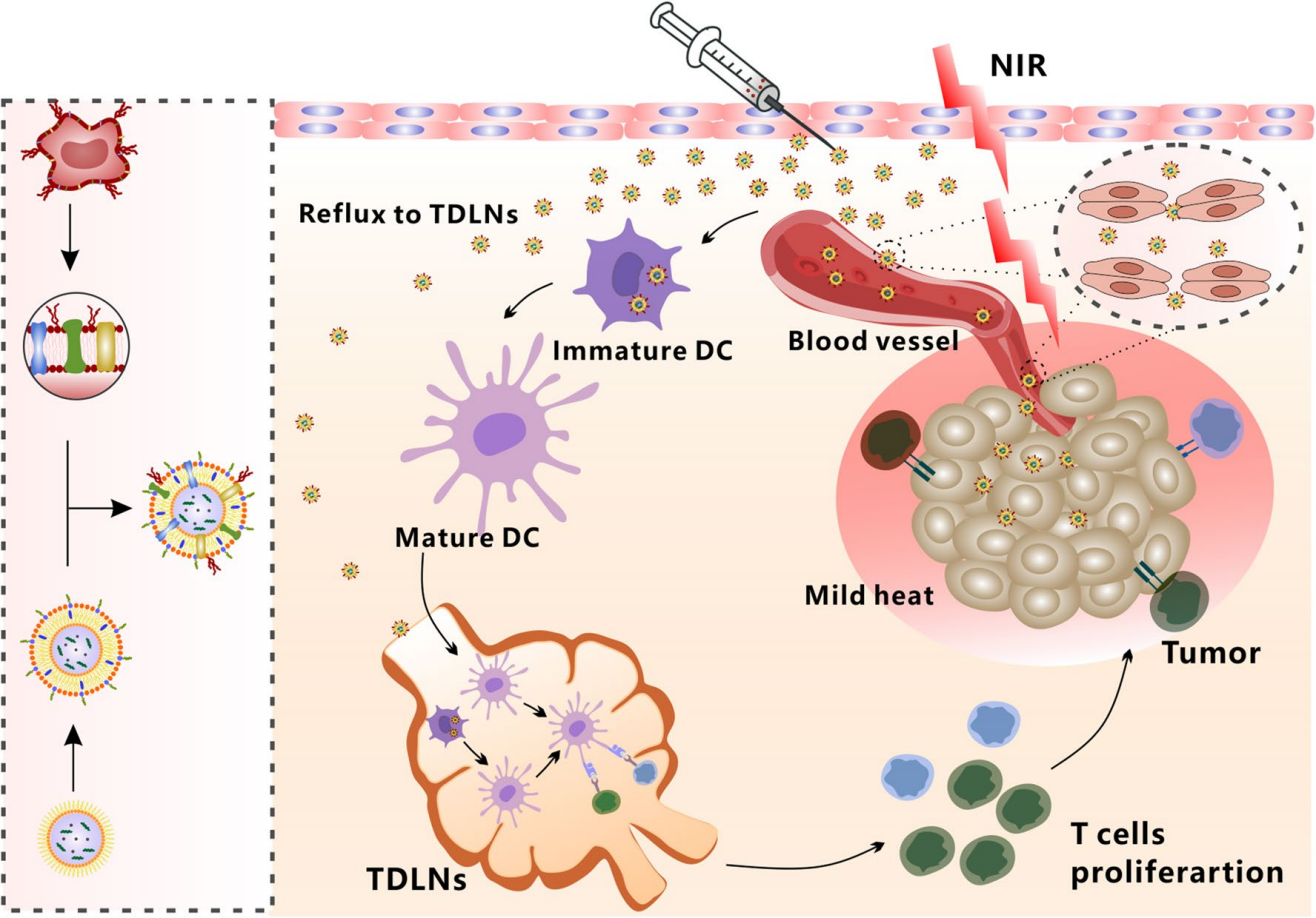
Schematic illustration of antitumor effect by DC hybrid nanovaccine

Here we prepared a kind of DC hybrid zinc phosphate nanoparticles (LDC@ZnP NPs) for cancer immunotherapy. The colon carcinoma (MC38) specific antigenic peptide Adpgk and photosensitizer melanin were co-loaded in zinc phosphate nanoparticles. The Zn^2+^ in nanoparticles can undergo special chelation with amino and phosphate groups of proteins so as to achieve efficient loading of antigen peptides [22]. Melanin is a natural biological pigment with strong near-infrared light absorption and can achieve photothermal conversion [23]. Meanwhile, it has high biocompatibility and metal ion chelating ability, which could also chelate with Zn^2+^ in nanoparticles [24, 25]. Lipid and DC membrane protein were further enveloped in the surface of nanoparticles to obtain the hybrid nanovaccine. This nanovaccine could actively target to DCs with the homologous proteins as DCs. When exposed to NIR laser, the nanovaccine achieved a satisfied mild photothermal effect. In addition, the vaccine can be effectively drained to the lymph nodes. After administration to MC38 tumor-bearing mice, the hybrid nanovaccines remarkably suppressed tumor growth by eliciting enhanced antitumor immunity (Scheme 1). Our work will provide a promising strategy for effective cancer immunotherapy.

## Results

### Preparation and characterization of LDC@ZnP NPs

**Fig. 1:**
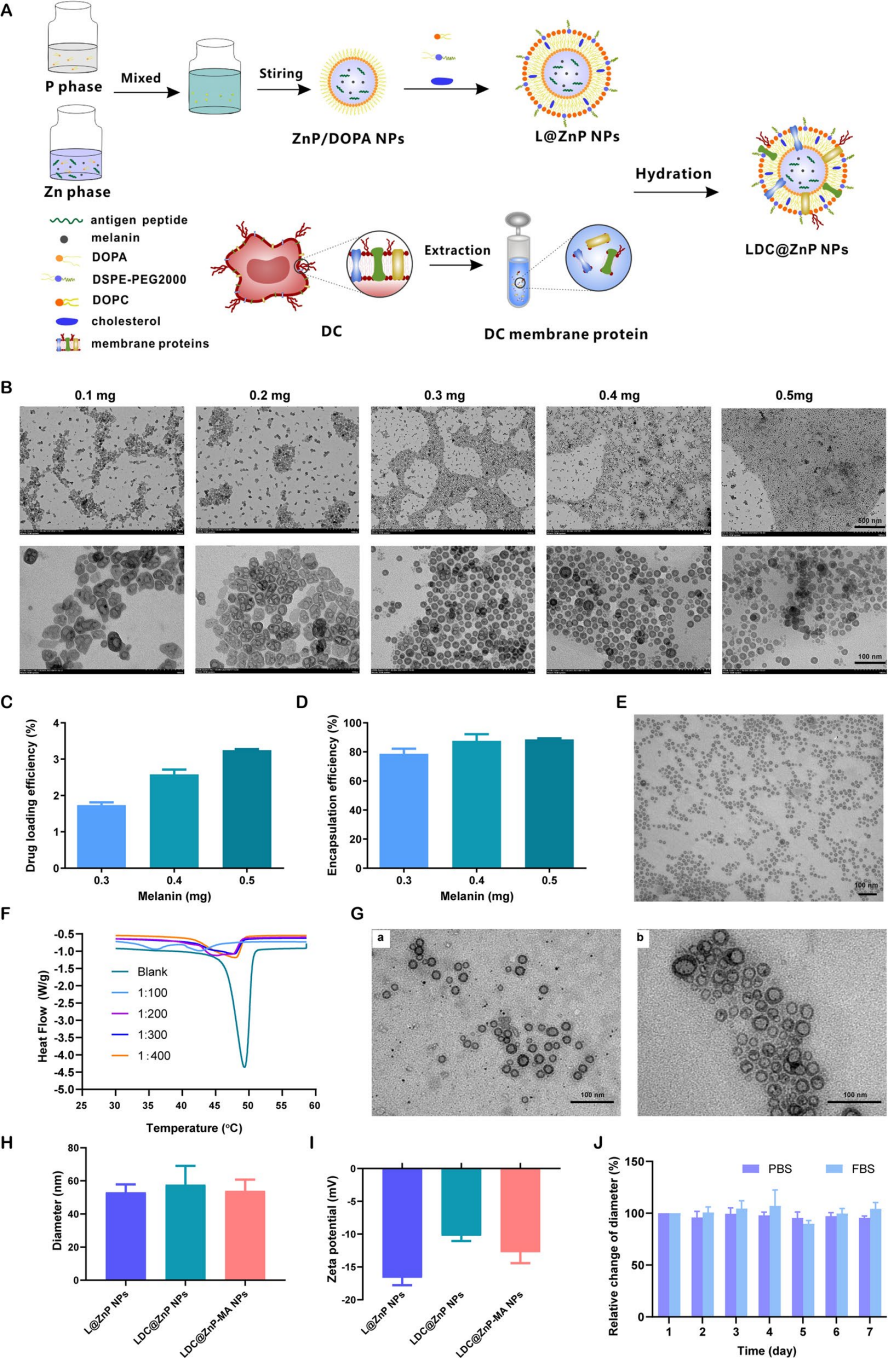
Preparation and characterization of LDC@ZnP NPs. **A** Schematic illustration for preparation of LDC@ZnP NPs. **B** TEM image of ZnP NPs with different amounts of melanin adding. The scale bar for the upper layer was 500 nm. The scale bar for the lower layer was 100 nm. **C** Drug loading efficiency by ZnP NPs with different amounts of melanin adding (n = 3). **D **Encapsulation efficiency by ZnP NPs with different amounts of melanin adding (n = 3). **E** TEM image of L@ZnP-M NPs, scale bar = 100 nm. F DSC curves of LDC@ZnP NPs with different amounts of DC membrane protein insertion. **G** TEM images of LDC@ZnP-M NPs (**a**) and LDC@ZnP-MA NPs (**b**), scale bar = 100 nm. **H** Hydration particle size of L@ZnP NPs, LDC@ZnP NPs and LDC@ZnP-MA NPs (n = 3). **I** Zeta potential of L@ZnP NPs, LDC@ZnP NPs and LDC@ZnP-MA NPs (n = 3). **J** Stability of LDC@ZnP NPs in PBS and FBS (n = 3)

The peptide and melanin co-loaded LDC@ZnP NPs were fabricated by reverse microemulsion combined with film-hydration method [22]. The schematic illustration of preparation was shown in Fig. 1A. The NPs formation mainly consisted of two steps: (1) preparation of separate Zn and P phase under the existence of DOPA in cyclohexane/Igepal CO-520 system. (2) formation of zinc phosphate precursors [26]. As shown in Additional file 1: Fig. S1, blank ZnP NPs displayed regular morphology and homogeneous distribution. To determine whether melanin impacted NP formation, different amount of melanin (0.1 mg, 0.2 mg, 0.3 mg, 0.4 mg and 0.5 mg) was added into the oil system of Zn phase, respectively. After interaction with P phase, the nanoparticles were collected by centrifugation, dispersed with chloroform and observed by TEM. As shown in Fig. 1B, when the amount of melanin was 0.3–0.5 mg, the nanoparticles presented regularly near-spherical with the optimal morphology at 0.4 mg. The loading efficiency of melanin by the nanoparticles was further determined. The drug loading efficiency increased with the increasing input of melanin (Fig. 1C). For the encapsulation efficiency, when the amount of melanin was 0.4 and 0.5 mg, both the encapsulation rate was around 90% (Fig. 1D). As the prepared ZnP NPs were hydrophobic, the NPs required hydrophilic modification for further application. Lipid envelopment is a commonly used method to modify the surface characteristics of nanoparticles to improve the stability and biocompatibility [27]. To obtain hydrophilic NPs, the ZnP NPs in chloroform were spun together with DOPC, cholesterol, and DSPE-PEG2000 (molar ratio 4:4:1) to form a thin film. After hydration, lipid-enveloped zinc phosphate nanoparticles (L@ZnP NPs) were obtained, which displayed similar morphology as ZnP NPs (Fig. 1E). The hydrophobic nanoparticles were transformed into nanoparticles that could disperse stably in aqueous solution by lipid envelopment, which solved the aggregation problem of ZnP NPs and was more conducive to the application of nanoparticles under physiological conditions.

To further functionalize L@ZnP NPs with dendritic cells, dendritic cell membrane proteins with different protein/phospholipid weight ratios (1:0, 1:100, 1:200, 1:300, 1:400) were added into PBS for hydration. Followed by an intense vortex for 3 min, ultrasound for 3 min, and incubation at 37 ^o^C water bath for 30 min, dendritic cell membrane protein hybrid zinc phosphate nanoparticles (LDC@ZnP NPs) were obtained. The protein profile of LDC@ZnP NPs was determined by sodium dodecyl sulfate polyacrylamide gel electrophoresis (SDS-PAGE). As shown in Additional file 1: Fig. S2, the protein in LDC@ZnP NPs matched with the parent cells, indicating the successful incorporation of DC membrane proteins in LDC@ZnP NPs. And the major membrane protein components could involve integral or lipid-anchored plasma membrane, cytosolic, cytoskeletal, peripheral, and secreted proteins [28, 29]. Then, the nanoparticles with different amounts of dendritic cell membrane proteins were freeze-dried and weighed. The corresponding temperature rise curve was obtained by a differential scanning calorimeter (DSC). As shown in Fig. 1F, when the protein/phospholipid weight ratio was 1:300 and 1:400, the nanosystem formed a single peak, indicating that cell membrane proteins were uniformly inserted into the phospholipid layer on the surface of the nanoparticles. For a larger amount of dendritic cell membrane protein insertion, 1:300 was chosen to prepare the dendritic cell membrane protein hybrid nanovaccine. TEM was further used to observe the membrane protein hybrid nanoparticles. As shown in Fig. 1G (a), the insertion of membrane proteins did not affect the morphology of melanin-loaded hybrid nanoparticles (LDC@ZnP-M NPs). Then the antigen peptide Adpgk and melanin co-loaded dendritic cell hybrid nanoparticles (LDC@ZnP-MA NPs) were prepared by the similar method. When the drug-fed amounts were 0.4 mg melanin and 0.25 mg peptide, the encapsulation efficiency of melanin and peptide by nanovaccine were 87.6 ± 4.6% and 57.2 ± 10.0%, respectively. The morphology of drug co-loaded hybrid nanoparticles showed a nearly spherical distribution as displayed in Fig. 1G (b).

Then the hydration particle size and zeta potential of nanoparticles were measured by DLS. As shown in Fig. 1H, I, compared with L@ZnP NPs, the particle size and potential values of blank LDC@ZnP NPs and drug-coloaded LDC@ZnP-MA NPs were slightly increased. The particle size was around 30 nm while the zeta potential was about − 10 mV. The stability of nanoparticles in PBS and FBS was further monitored by DLS, and there was no significant difference in the change of particle size during one week (Fig. 1J).

### The in vitro photothermal effect of LDC@ZnP-M NPs

**Fig. 2:**
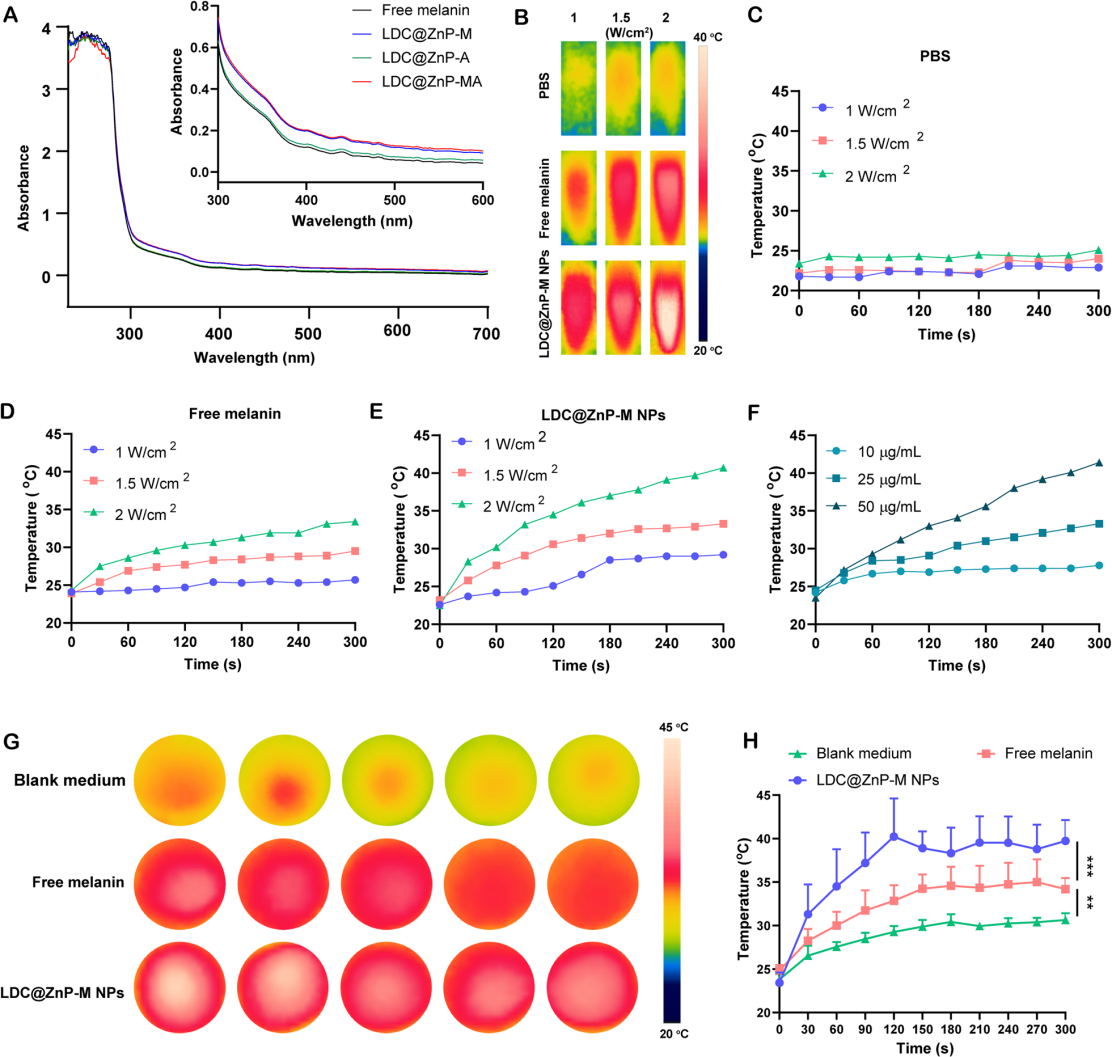
Photothermal effect of LDC@ZnP NPs. **A** UV absorption spectra of free melanin and different nanoparticles. **B** Photothermal imaging of PBS, free melanin (50 µg/mL) and equivalent LDC@ZnP-M NPs under NIR irradiation for 5 min with different irradiation powers. **C**–**E** Photothermal profiles of PBS (**C**), free melanin (**D**) and LDC@ZnP-M NPs (**E**). **F** Photothermal profiles of LDC@ZnP-M NPs with different concentrations. **G** Photothermal images of blank medium, free melanin and LDC@ZnP-M NPs (n = 5). **H** Photothermal profiles of blank medium, free melanin and LDC@ZnP-M NPs (n = 5), **p < 0.01, ***p < 0.001

Firstly, UV absorption of free melanin, Adpgk loaded dendritic cell hybrid nanoparticles (LDC@ZnP-A NPs), LDC@ZnP-M NPs, and LDC@ZnP-MA NPs was detected by UV-VIS absorption spectroscopy, respectively. Free melanin is poorly soluble in PBS while LDC@ZnP-M NPs and LDC@ZnP-MA NPs enhanced the soluble status of melanin by nanoparticle entrapment. And the enhanced solubility is correlated with the absorption in UV spectra [30]. As a result, LDC@ZnP-M NPs and LDC@ZnP-MA NPs showed similarly stronger absorption in the near infrared region than free melanin and LDC@ZnP-A NPs (Fig. 2A). Then to monitor the photothermal effect under NIR irradiation, PBS, Free melanin and equivalent LDC@ZnP-M NPs were exposed to 808 nm laser at different laser powers. The real-time temperature of each group was recorded by infrared thermal imaging camera. As shown in Fig. 2B–E, PBS showed negligible temperature change under different power. Free melanin and LDC@ZnP-M NPs showed enhanced photothermal effect with the increase of irradiation power and time. Notably, LDC@ZnP-M NPs displayed more remarkable temperature increase than free melanin. Among the three irradiated powers, 2 W/cm^2^ irradiation could achieve a satisfied mild-heat effect of LDC@ZnP-M NPs and was thereby selected for follow-up experiments. Then the nanoparticles were diluted into a series of concentrations with PBS and irradiated with 2 W/cm^2^ laser. As shown in Fig. 2F, the photothermal effect of LDC@ZnP-M NPs was in a dose-dependent manner. Moreover, the photothermal effect of LDC@ZnP-MA NPs (50 µg/mL melanin, 20 µg/mL Adpgk) was monitored under 2 W/cm^2^ irradiation for 5 min. A shown in Additional file 1: Fig. S3, LDC@ZnP-MA NPs displayed similar profile with LDC@ZnP-M NPs, indicating that the peptide loading showed negligible impact on photothermal effect of NPs. Then the intracellular photothermal effect of LDC@ZnP-M NPs was investigated. BMDCs were treated with blank medium, free melanin (50 µg/mL) and equivalent LDC@ZnP-M NPs, respectively. After 4 h, the supernatant was discarded and PBS was added. The plate was placed at room temperature and irradiated with 808 nm laser at 2 W/cm^2^. Temperatures were recorded at different time points (0 s, 30 s, 60 s, 90 s, 120 s, 150 s, 180 s, 210 s, 240 s, 270 s, 300 s). As Fig. 2G, H showed, compared with blank medium and free melanin, LDC@ZnP-M NPs increased rapidly within 120 s and then maintained at about 42 ℃, showing a good intracellular mild photothermal effect.

### The in vitro interaction of LDC@ZnP NPs and BMDCs

**Fig. 3:**
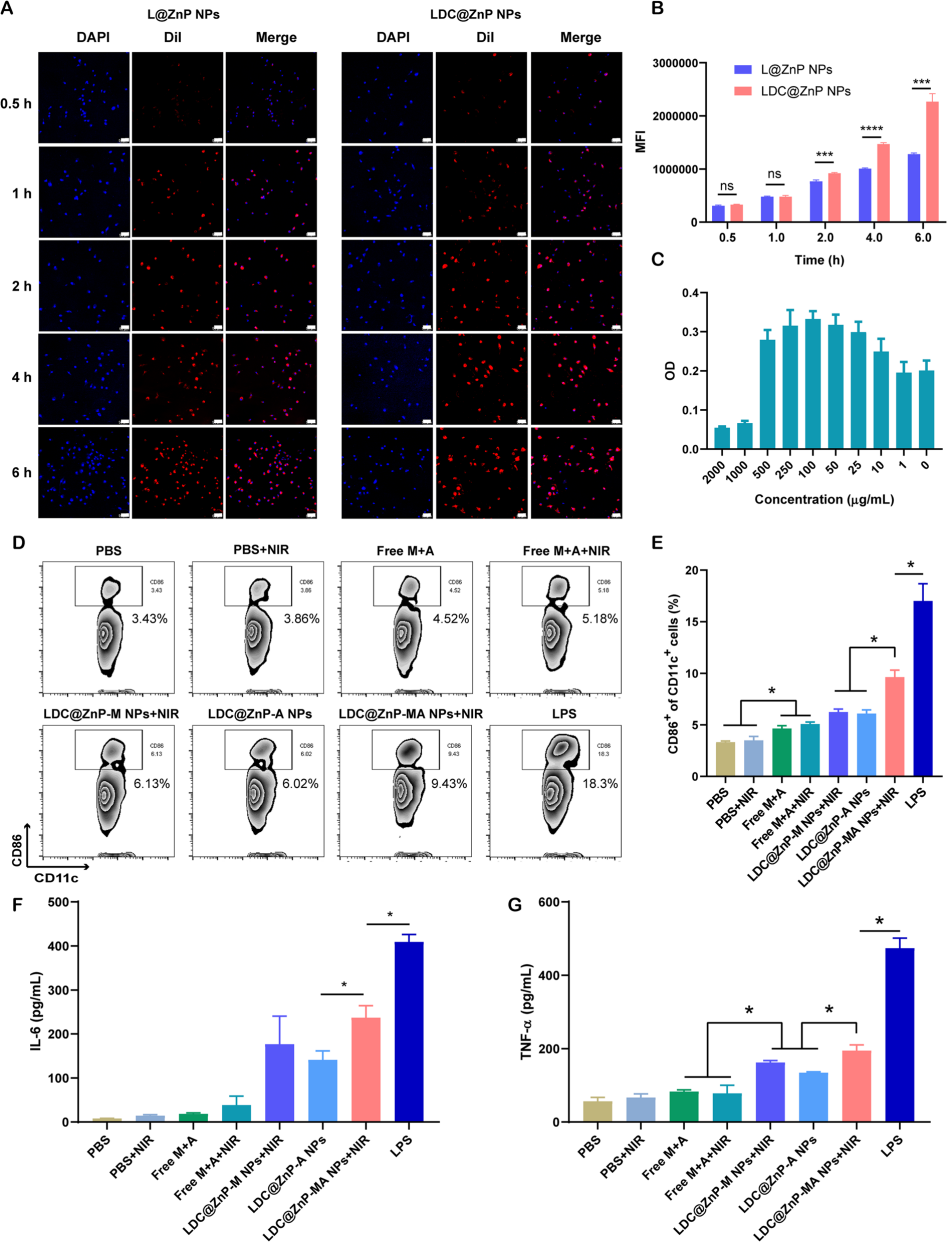
The in vitro interaction of LDC@ZnP NPs and BMDCs. **A** Confocal images of BMDCs after incubation with L@ZnP NPs and LDC@ZnP NPs. Cell nuclei were stained with DAPI, scale bar = 50 μm. **B **Quantitative uptake of L@ZnP NPs and LDC@ZnP NPs by BMDCs detected by flow cytometer (n = 3), ns: not significant, ***p < 0.001, ****p < 0.0001. **C** The OD values of BMDCs incubated with different amounts of LDC@ZnP-MA NPs (n = 4). **D **Flow scatter plots of CD11c^+^CD86^+^ cells. **E** Quantitative analysis of CD11c^+^CD86^+^ cells (n = 3), *p < 0.05. **F** The level of IL-6 in the supernatant of BMDCs (n = 3), *p < 0.05. **G **The level of TNF-α in the supernatant of BMDCs (n = 3), *p < 0.05

L@ZnP NPs and LDC@ZnP NPs were labeled with fluorescent dye DiI [31], and incubated with BMDCs for 0.5, 1, 2, 4, and 6 h, respectively, to determine the internalization behaviors of NPs. To visualize the intracellular uptake, the cells were stained with DAPI and observed by confocal microscope. As shown in Fig. 3A, the uptake of L@ZnP and LDC@ZnP NPs by DCs was time-dependent. Within a relatively short period of 0.5 and 1 h, homologous targeting of LDC@ZnP NPs to DCs was not obvious. With incubation time prolonging, the uptake of LDC@ZnP NPs by DCs was remarkably enhanced than that of L@ZnP NPs. Furthermore, flow cytometry was used for quantitative analysis of cell uptake. It showed a similar tendency as the results of confocal images (Fig. 3B). These results indicated that the hybrid of DC membrane proteins to L@ZnP NPs could achieve the homologous targeting effect to DCs. It could be attributed to the molecule-mediated homotypic cell surface interactions, which would promote intracellular antigen accumulation in DCs [32].

After uptake antigen, immature DCs need to undergo maturation for antigen presentation [33]. Here, the cytotoxicity of LDC@ZnP-MA NPs to BMDCs was firstly investigated before conducting other assays. LDC@ZnP-MA NPs were diluted into a series of concentrations (NPs amount) with medium. As displayed in Fig. 3C, LDC@ZnP-MA NPs caused toxicities to BMDCs at high doses of 1000 and 2000 µg/mL. When the dose was lower than 500 µg/mL, the NPs were safe for DCs. Notably, DCs were proliferative at the doses varying from 10 to 500 µg/mL compared to the control group, suggesting that LDC@ZnP-MA NPs promoted the proliferation of BMDCs. It could be attributed to that the stimulation to DC promotes the expansion of DCs [34, 35]. Then the photothermal safety was investigated when combined with NIR irradiation. 5 µg/mL and 10 µg/mL of LDC@ZnP-M NPs (corresponding to 193 and 385 µg/mL NPs) were used to incubate with BMDCs. After co-incubating for 4 h, the supernatant was discarded and replaced with fresh medium. Then the cells were placed on the thermostat controlled hot plate (37 ℃) to maintain the cell viability, irradiated with 808 nm laser for different time and continued to culture for another 20 h. After irradiating for 2 min, the temperature of 5 µg /mL and 10 µg /mL LDC@ZnP-M NPs (melanin amount) treated cells were 38.9 ± 0.6 ℃ and 41.2 ± 0.8 ℃, respectively, which were no more than the cytotoxic temperature above 42 ℃. As shown in Additional file 1: Fig. S4, both 5 µg /mL and 10 µg /mL LDC@ZnP-M NPs showed good photothermal safety compared to blank medium. Notably, the cell viabilities of LDC@ZnP-M NPs treated cells were remarkably higher than blank medium, which could be attributed to the immune stimulation of LDC@ZnP-M NPs to DCs. Moreover, to determine the stimulation of LDC@ZnP-MA NPs on DC maturation, BMDCs were incubated with PBS+/-NIR, Free M + A+/-NIR (10 µg/mL melanin, 4 µg/mL Adpgk), equivalent LDC@ZnP-M NPs + NIR (corresponding to 385 µg/mL NPs), LDC@ZnP-A NPs, LDC@ZnP-MA NPs + NIR and LPS (1 µg/mL) for 4 h and replaced with fresh medium. For the groups with NIR irradiation, the cells were exposed to 2 W/cm^2^ laser for 2 min. After another 20 h, the cells were collected and stained with DC (CD11c) and maturation maker (CD86) and detected by flow cytometry. As shown in Fig. 3D, E, LPS can significantly up-regulate the proportion of CD11c^+^CD86^+^ matured DCs. Compared with Free M + A+/-NIR, LDC@ZnP-M NPs + NIR mediated photothermal therapy or LDC@ZnP-A NPs-mediated immunotherapy, LDC@ZnP-MA NPs + NIR-mediated combinational therapy had a significant stimulatory effect on DC maturation. Accompany with maturation process, DCs are prone to secret immunostimulatory cytokines to the extracellular environment. Here the supernatant was centrifuged to get rid of the cells and debris and measured by ELISA kits. Interleukin 6 (IL-6) is obligated to induce T cell-mediated autoimmunity [36]. Tumor necrosis factor-alpha (TNF-α) is a mediator in cellular immunity, which could promote the cross-presentation by DCs [37]. As shown in Fig. 3F, G, secretion levels of IL-6 and TNF-α were remarkably enhanced in LDC@ZnP-MA NPs + NIR treated groups. The results indicated that LDC@ZnP NPs can be effectively uptake and processed by DCs.

### Lymphatic reflux of LDC@ZnP NPs

**Fig. 4:**
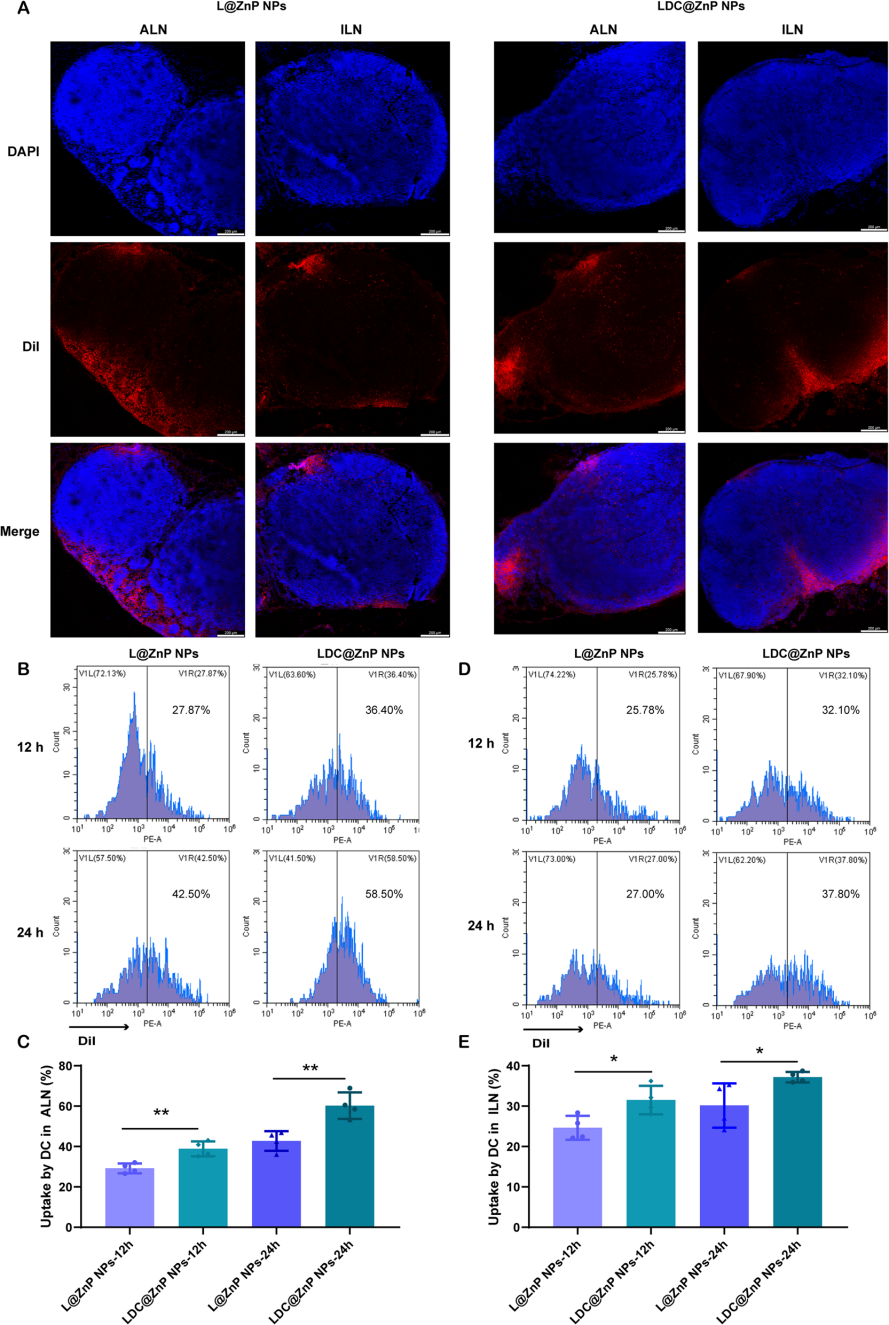
Lymphatic reflux of LDC@ZnP NPs. **A** Distribution of L@ZnP NPs and LDC@ZnP NPs in ALNs and ILNs, scale bar = 200 μm. **B** Uptake of L@ZnP NPs and LDC@ZnP NPs by DCs in ALNs. **C** Quantitative analysis of L@ZnP NPs and LDC@ZnP NPs uptake behaviors by DCs in ALNs (n = 4), **p < 0.01. **D **Uptake of L@ZnP NPs and LDC@ZnP NPs by DCs in ILNs. **E** Quantitative analysis of L@ZnP NPs and LDC@ZnP NPs uptake behaviors by DCs in ILNs (n = 4), *p < 0.05

It has been evidenced that the in vivo biodistribution of nanoparticles is related with properties of nanoparticles including the elemental-chemical compositions, size distribution, shape, surface hydrophobicity, agglomeration state, optical properties, etc. [38, 39]. Generally, the drug loading in the core of nanoparticles with core-shell structure has negligible influence to these properties, leading to the similar biodistribution behavior of drug-loaded nanoparticles with blank nanoparticles. Herein, L@ZnP NPs and LDC@ ZnP NPs were used to monitor the in vivo biodistribution. TDLNs play a significant role in cancer immunotherapy, especially for vaccines. As inguinal and axillary lymph nodes are the most important TDLNs after subcutaneous injection of nanovaccine [40], DiI labeled L@ZnP and LDC@ZnP NPs were then injected subcutaneously into the axillary and inguinal regions of C57BL/6 mice, respectively. After 24 h, the mice were sacrificed for the collection of ALNs and ILNs. The tissues were prepared for frozen sections and stained with DAPI. As shown in Fig. 4A and Additional file 1: Fig. S5, the red fluorescence of LDC@ZnP NPs was stronger in both ALNs and ILNs when compared with L@ZnP NPs. Interestingly, for both L@ZnP NPs and LDC@ZnP NPs, the fluorescence intensity and distribution in the ALNs were enhanced than those in ILNs. To further evaluate the cell uptake behavior by DCs in LNs, ALNs and ILNs were collected for flow cytometry analysis after subcutaneous administration of DiI labeled LZnP and LDC@ZnP NPs. At 12 and 24 h post-injection, the lymphocytes in LNs were stained with CD11c. As shown in Fig. 4B–E, with the extension of injection time, the uptake of both nanoparticles by DCs were obviously increased. Compared with DCs in ILN, DCs in ALN uptake more L@ZnP NPs and LDC@ZnP NPs. Similar to the in vitro uptake behavior by BMDCs, LDC@ZnP NPs showed enhanced internalization by both ALN and ILN DCs than LZnP NPs. In combination with the distribution and uptake of LDC@ZnP NPs, ALNs could be more suitable as the tumor-draining lymph nodes.

### Biodistribution of LDC@ZnP NPs

**Fig. 5:**
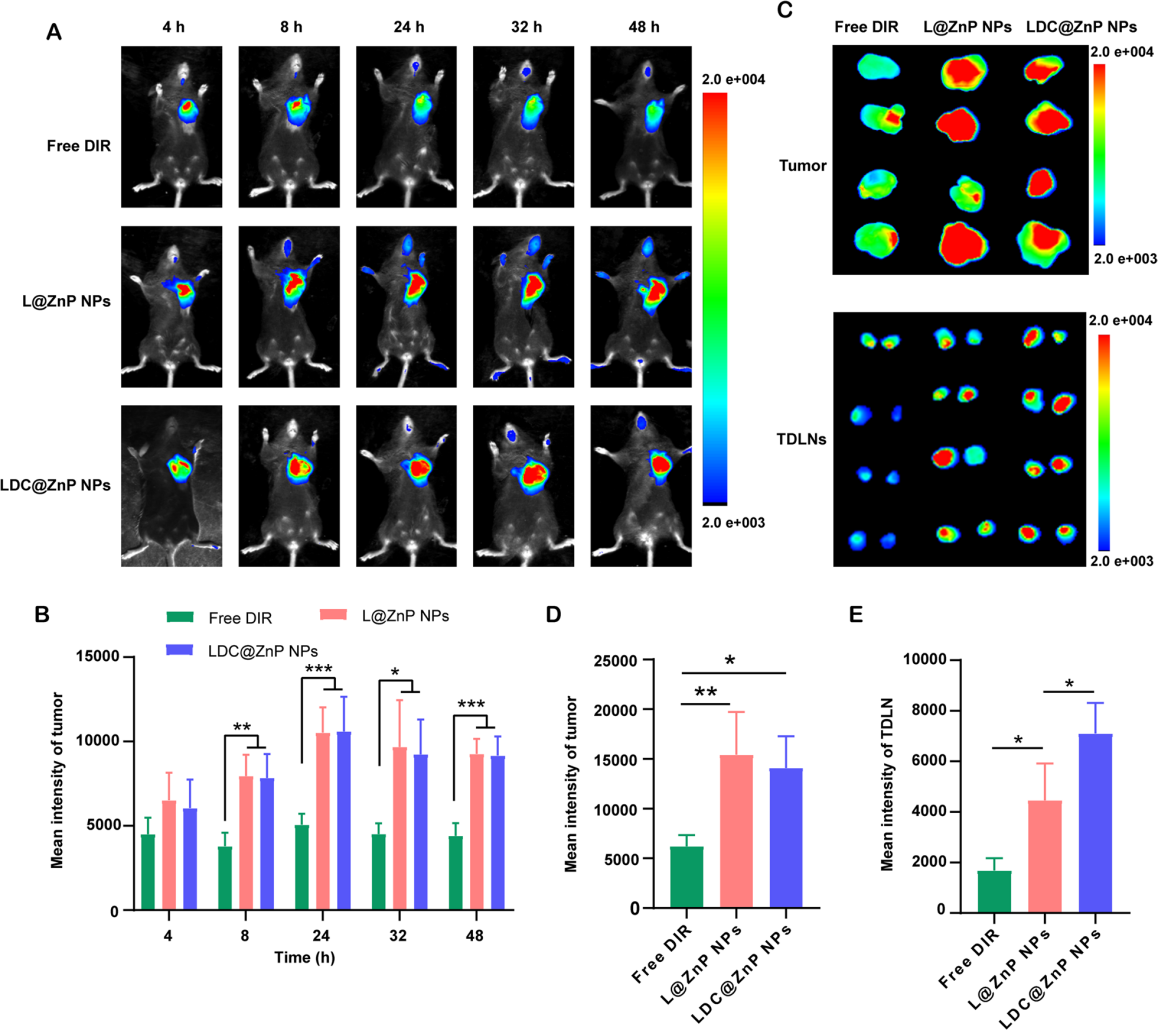
Biodistribution of LDC@ZnP NPs in vivo. **A** In vivo images of Free DIR, L@ZnP NPs and LDC@ZnP NPs treated mice at different time points. **B** Fluorescence quantization of in vivo tumors at different time points (n = 4), *p < 0.05, **p < 0.01, ***p < 0.001. **C** Ex vivo images of Free DIR, L@ZnP NPs, and LDC@ZnP NPs treated tumors and TDLNs at 48 h post injection (n = 4). **D** Fluorescence quantization of ex vivo tumors at 48 h post injection (n = 4), *p < 0.05, **p < 0.01. **E** Fluorescence quantitation of Free DIR, L@ZnP NPs and LDC@ZnP NPs in ex vivo TDLNs (n = 4), *p < 0.05

MC38 cells were inoculated at the right flank of female C57BL/6 mice. When the tumor volume reached around 500 mm^3^, the mice were randomly divided into three groups. The near-infrared dye DIR, DIR-labeled L@ZnP or LDC@ZnP NPs were injected subcutaneously to the tumor-bearing mice to track the in vivo biodistribution. Fluorescence images were monitored at different time points (4, 8, 24, 32, 48 h). Different with healthy blood vessels, the blood vessels surrounding tumor are usually discontinuous and irregular owing to the excessive secretion of angiogenic factors by tumor. These malformed blood vessels are lack of basement membrane, thereby displaying increased permeability with enlarged intercellular gaps [41]. Moreover, a dynamic blood vessel bursts happened in tumor blood vessels, which allowed the outflow fluid extravasate into the interstitial space of tumor [42]. As shown in Fig. 5A, the distribution of L@ZnP NPs and LDC@ZnP NPs in tumors increased during 4 to 24 h, reached a peak at 24 h, and maintained a relatively strong fluorescence intensity at 48 h. Fluorescence quantitative results showed that there was no significant difference in the fluorescence intensity between L@ZnP NPs and LDC@ZnP NPs in tumors (Fig. 5B). The delivery of NPs to tumor could be attributed to the formation of large-size gaps as well as the dynamic vascular vent in tumor blood vessels. At 48 h post-injection, the mice were sacrificed. The tumor tissues and TDLNs were stripped for ex vivo near-infrared imaging. As shown in Fig. 5C, D, the ex vivo fluorescence of tumor was consistent with the in vivo results. Notably, LDC@ZnP NPs treated group showed increased fluorescence intensity in TDLNs than L@ZnP NPs treated group (Fig. 5E), further indicating the lymphatic reflux effect of LDC@ZnP NPs in vivo. Moreover, the major tissues, including heart, liver, spleen, lung and kidney, were also dissected. As presented in Additional file 1: Fig. S6, Free DIR, L@ZnP NPs and LDC@ZnP NPs were distributed in all tissues while showing a major distribution in the livers with rich blood supply, indicating that LDC@ZnP NPs can be absorbed subcutaneously into the bloodstream.

### In vivo antitumor effect mediated by LDC@ZnP NPs

**Fig. 6:**
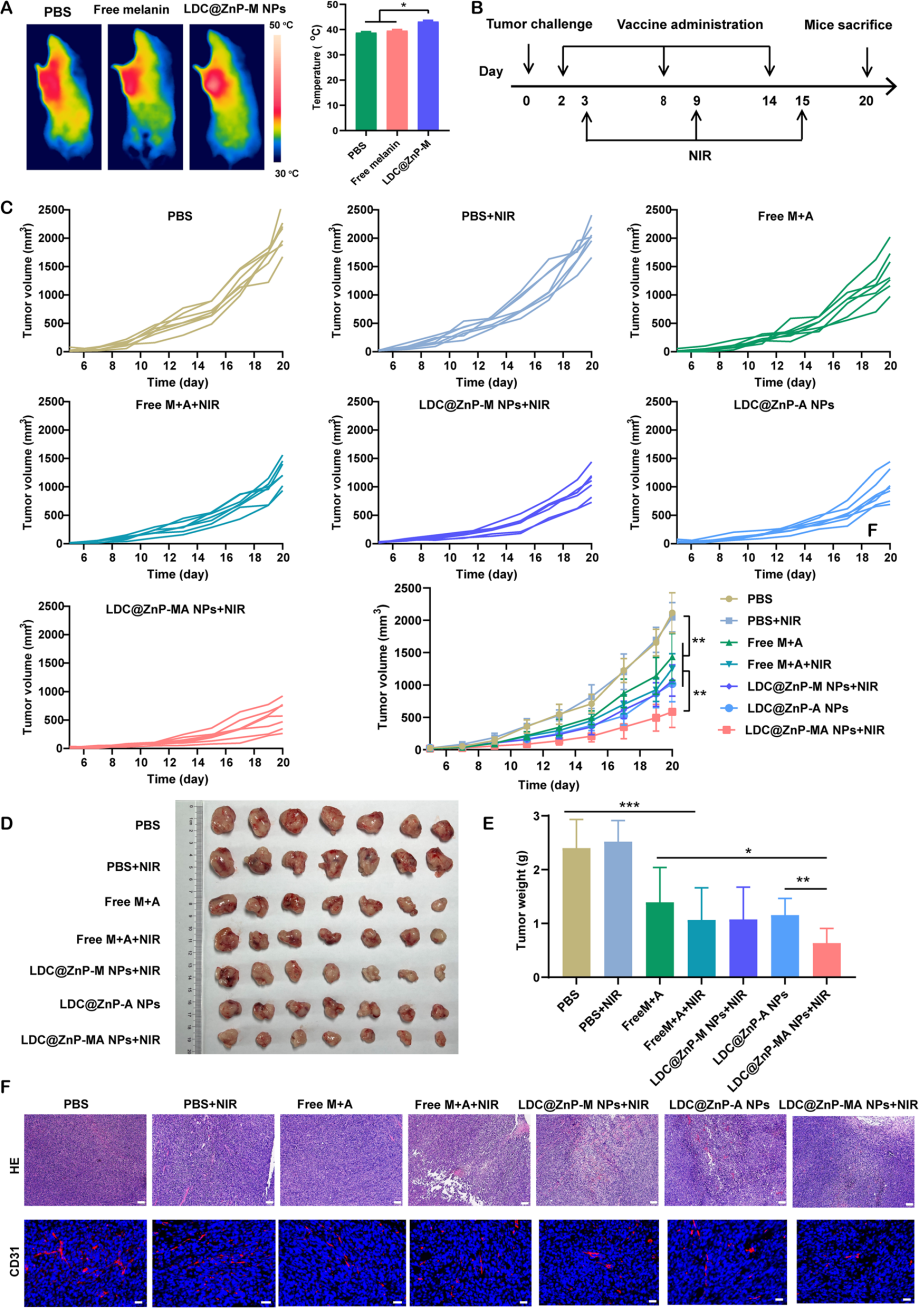
In vivo antitumor effects of LDC@ZnP NPs. **A** The in vivo photothermal images (left) and temperature (right) after irradiating with 808 nm laser for 2 min (n = 3), *p < 0.05. **B** Time schedule of treatment. **C** Tumor growth curves of MC38 treated by different formulations (n = 7), *p < 0.05, **p < 0.01. **D** Images of dissected tumors (n = 7). **E** Tumor weight in each group (n = 7), *p < 0.05, **p < 0.01, ***p < 0.001. **F** HE and CD31 staining images of tumor tissue after different treatments, scale bars were 200 nm for HE and 20 nm for CD31 staining, respectively

Prior to investigating the antitumor efficiency of LDC@ZnP NPs, the in vivo photothermal effect was determined in tumor-bearing C57BL/6 mice. The mice were administered with PBS, Free melanin, and LDC@ZnP-M NPs. After 24 h, the mice in each group were anesthetized with pentobarbital sodium and irradiated with 808 nm laser. The temperature of LDC@ZnP-M NPs treated tumors could increase to around 42 ^o^C within 2 min while that of PBS and Free melanin were lower than 40 ^o^C **(**Fig. 6A**)**. To further determine the impact of peptide coloading on the heating effect of NPs, the in vivo photothermal effect of LDC@ZnP-MA NPs were evaluated after subcutaneous injection. The corresponding amounts of melanin and Adpgk peptide were 50 µg and 20 µg, respectively. As Additional file 1: Fig. S7 displayed, LDC@ZnP-MA NPs can achieved approximately 42 ℃ after irradiation for 2 min (2 W/cm^2^), which were similar as LDC@ZnP-M NPs. Then the in vivo antitumor effect of different treatments was investigated. 1.25 × 10^5^ MC38 cells were inoculated into the right frank of C57BL/6 mice. PBS, PBS + NIR, Free M + A (50 µg melanin and 20 µg Adpgk), Free M + A + NIR, LDC@ZnP-M NPs + NIR, LDC@ZnP-A NPs, LDC@ZnP-MA NPs + NIR were given to the tumor-bearing mice, respectively. The vaccination and irradiation followed the protocol in Fig. 6B. The mental and physiological conditions as well as body weight were monitored. During the therapeutic period, the body weight of all treated mice showed an increased tendency (Additional file 1: Fig. S8A), indicating the biosafety of LDC@ZnP NPs. Meanwhile, tumor growth was monitored by recording the width and length of tumor. As presented in Fig. 6C, the tumor growth of PBS + NIR treated mice were similar with PBS, demonstrating the photothermal safety of NIR in vivo. Compared with Free M + A and Free M + A + NIR treated mice, the antitumor effect of LDC@ZnP NPs was more obvious. LDC@ZnP-M NPs + NIR or LDC@ZnP-A NPs mediated immunotherapy displayed comparable tumor suppression. Notably, the combination of peptide and mile heat inspired immunotherapy mediated by LDC@ZnP-MA NPs + NIR showed the best tumor inhibition performance among all treatments. Consisting with the results of tumor volume, the excised tumor image (Fig. 6D) and tumor mass (Fig. 6E) also evidenced the effective antitumor activity of LDC@ZnP-MA NPs. Furthermore, the tumor inhibitory rates were calculated by contrast with PBS. Accordingly, LDC@ZnP-MA NPs treated mice presented the highest tumor inhibitory rate of 73.5% (Additional file 1: Fig. S8B). Afterwards, HE and CD31 immunofluorescent staining of tumor sections was conducted to better explore the antitumor outcomes of LDC@ZnP NPs. As displayed with the largest areas of tumor lysis and necrosis, LDC@ZnP-MA NPs showed superior therapeutic effect among all treatments. CD31, highly expressed on endothelial cells, is well established to monitor the vessel density in malignant tissues [43]. As shown in Fig. 6F, a reduction of CD31 expression was observed in LDC@ZnP NPs treated tumors.

### The antitumor immune responses elicited by LDC@ZnP NPs

**Fig. 7:**
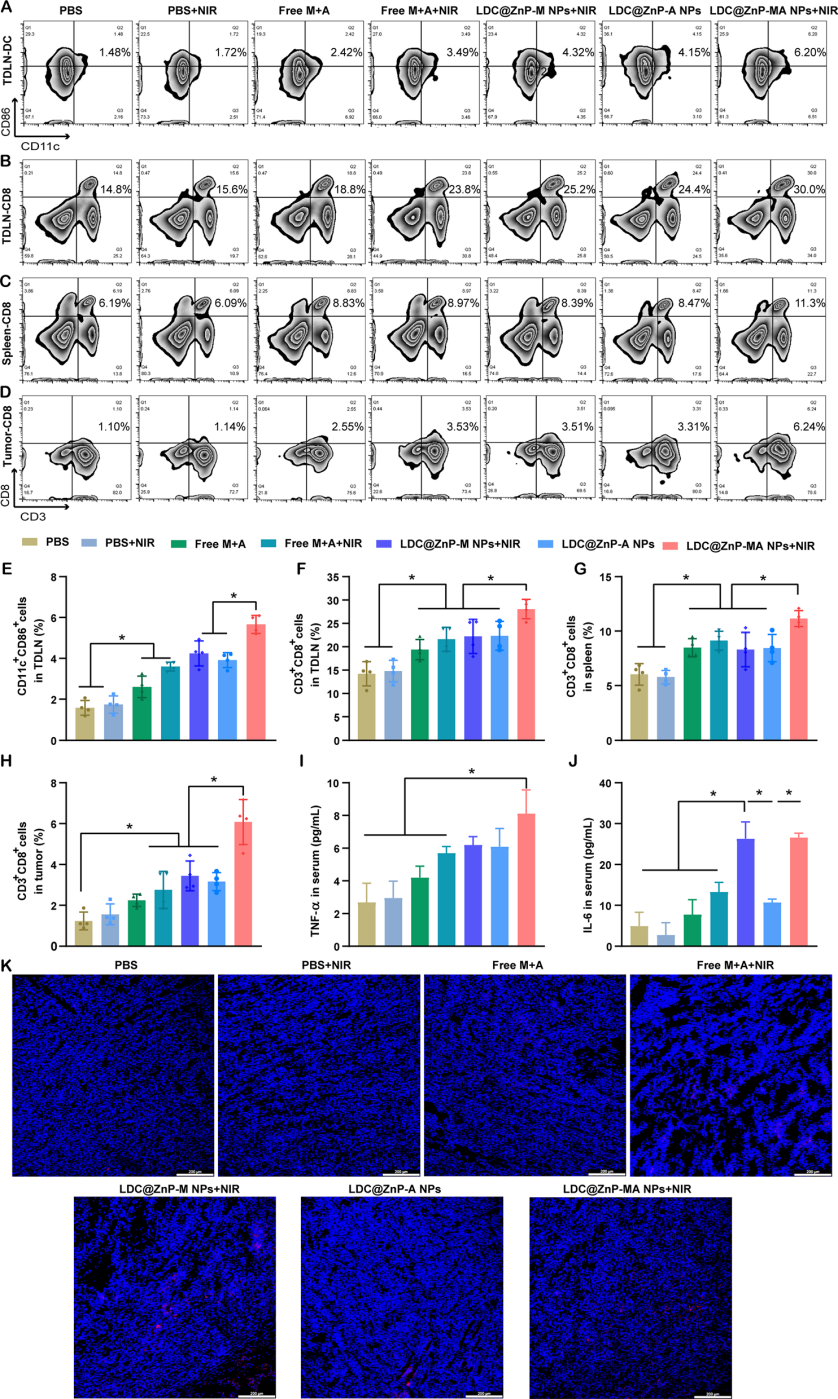
The antitumor immune responses elicited by LDC@ZnP NPs. The mice were treated with PBS, PBS + NIR, Free M + A, Free M + A + NIR, LDC@ZnP-M NPs + NIR, LDC@ZnP-A NPs and LDC@ZnP-MA NPs + NIR, respectively. **A** Flow scatter plots of CD11C^+^CD86^+^ cells in TDLNs. **B** Flow scatter plots of CD3^+^CD8^+^ cells in TDLNs. **C** Flow scatter plots of CD3^+^CD8^+^ cells in spleens. **D** Flow scatter plots of CD3^+^CD8^+^ cells in tumor tissues. **E** Quantitative analysis of CD11C^+^CD86^+^ cells in TDLNs (n = 4), *p < 0.05. **F** Quantitative analysis of CD3^+^CD8^+^ cells in TDLNs (n = 4), *p < 0.05. **G** Quantitative analysis of CD3^+^CD8^+^ cells in spleens (n = 4), *p < 0.05. **H** Quantitative analysis of CD3^+^CD8^+^ cells in tumors (n = 4), *p < 0.05. **I **Serum levels of TNF-α (n = 3), * *p* < 0.05. **J** Serum levels of IL-6 (n = 3), *p < 0.05. **K** Immunofluorescent staining of IL-6 in tumor sections. The nuclei were stained with DAPI, scale bar = 200 μm

To explore the underlying mechanism of LDC@ZnP NPs mediated immunotherapy, we analyzed the immune cells and cytokines after 48 h of last injection. TDLNs are correlated with antitumor immunity due to the specific location and roles. We have evidenced that LDC@ZnP NPs can effectively reflux to TDLNs and be uptake by DCs. As DC maturation was essential for antigen presentation, here we firstly analyzed the in vivo DC maturation after vaccination by different formulations. As shown in Fig. 7A and E, the matured DCs in PBS + NIR, Free M + A, Free M + A + NIR, LDC@ZnP-M NPs + NIR, LDC@ZnP-A NPs, LDC@ZnP-MA NPs + NIR treated TDLNs were 1.1-, 1.7-, 2.3-, 2.7-, 2.5- and 3.6-fold compared with PBS, respectively. Notably, LDC@ZnP-MA NPs + NIR presented the highest DC maturation among all groups, which would be beneficial to induce subsequent antitumor immune response. As an important lymphoid tissue for systematic antitumor immunity, the DC maturation of spleen was also analyzed, which showed similar results as TDLNs (Additional file 1: Fig. S9). It was also found that DC infiltrated in tumor microenvironment, and LDC@ZnP-MA NPs + NIR treated groups increased DC population compared with the control group (Additional file 1: Fig. S10). After maturation, DCs would present the tumor associated antigens to T cells to elicit specific antitumor immunity [44]. As a crucial component of T cells, cytotoxic lymphocytes (CTLs, CD3^+^CD8^+^ cells) play an essential role in antitumor immunotherapy [45, 46]. Here, we analyzed CTLs in TDLNs, spleen, and tumor tissues. In TDLNs, the proportion of CTLs for PBS, PBS + NIR, Free M + A, Free M + A + NIR, LDC@ZnP-M NPs + NIR, LDC@ZnP-A NPs and LDC@ZnP-MA NPs + NIR were 14.2 ± 2.6%, 14.8 ± 2.36%, 19.4 ± 2.2%, 21.6 ± 2.6%, 22.2 ± 3.7%, 22.3 ± 3.1% and 28.1 ± 2.1%, respectively (Fig. 7B, F). Meanwhile, CTLs in spleen of PBS, PBS + NIR, Free M + A, Free M + A + NIR, LDC@ZnP-M NPs + NIR, LDC@ZnP-A NPs and LDC@ZnP-MA NPs + NIR treated mice were 6.0 ± 1.0%, 5.8 ± 0.6%, 8.5 ± 0.8%, 9.1 ± 0.9%, 8.3 ± 1.6%, 8.4 ± 1.2% and 11.2 ± 0.7%, respectively (Fig. 7C and G). For tumor microenvironment, the infiltration of CD3^+^CD8^+^ cells in LDC@ZnP-MA NPs + NIR treated group was 4.9-, 3.9-, 2.7-, 2.2-, 1.8-, and 1.9-fold than in PBS, PBS + NIR, Free M + A, Free M + A + NIR, LDC@ZnP-M NPs + NIR, LDC@ZnP-A NPs treated groups, respectively (Fig. 7D and H). CD4^+^ helper T cells, an important subset of T cells, promote both the effector and memory functions of CTLs and help CTLs to overcome negative regulations [47]. After vaccination by LDC@ZnP-MA NPs + NIR, the percentage of CD3^+^CD4^+^ cells were remarkably enhanced compared with other treatments (Additional file 1: Fig. S11, S12). Moreover, the release of immunostimulatory cytokines after different vaccinations was determined by ELISA. As presented in Fig. 7I, J, the serum levels of TNF-α and IL-6 showed the highest secretion in LDC@ZnP-MA NPs + NIR treated mice among all the groups. The immunofluorescent staining of IL-6 in tumor sections displayed similar results (Fig. 7K). These results suggested that the combination of tumor associated antigen and mild heat inspired immunotherapy could induce effective antitumor immune responses.

## Discussion

Cancer immunotherapy manipulates the immune system to selectively eliminate the malignant tumor cells, avoiding non-specific or adverse reactions to normal tissues. Therapeutic cancer vaccines emerge as a potential candidate in cancer immunotherapy, aiming to realize tumor regression, eradicate the minor residual lesion and maintain long-lasting immune memory effect [48]. However, tumor could undergo microevolution to evade the surveillance of immune system, posing significant challenges to achieve this goal. Recently, combinational immunotherapy, exerting multiple mechanisms to synergistically work in tumor suppression, has been wildly explored in cancer treatment [49, 50]. Here we fabricated DC hybrid ZnP nanoparticles to combine the tumor associated antigen mediated antitumor immunity with mild-heat inspired immunotherapy. The purified antigenic peptide was selected as the tumor associated antigen owing to the advantages of non-toxic, free of infection factors and directly being presented by APCs without intracellular processing [51]. These peptide-based vaccines undergo intracellular uptake and processing by professional antigen presenting cells. Followed with antigen presentation to T cells, peptide vaccines can evoke sustained T cell responses [52]. Moreover, Zn^2+^ in the formulated nanoparticles can chelate with amino and phosphate groups in peptides to effectively encapsulate the antigenic peptides [53]. Melanin, a natural dark pigment, can strongly absorb near-infrared light and convert the light into heat, making it a potential candidate for photothermal therapy. Besides, melanin can chelate with metal ions, which facilitates the loading by ZnP nanoparticles [54]. To achieve DC homologous targeting effect, the nanovaccine was further functionalized with DC membrane proteins. The obtained nanovaccine showed enhanced uptake behaviors by DCs in vitro and in vivo, which could be attributed to multiple molecule-mediated homotypic cell surface interactions between nanovaccine and DCs [55]. This kind of DC hybrid nanovaccine had a negative zeta potential of -10 mV and small particle size of around 30 nm. The negative charge facilitated the in vivo physiological stability and biological safety of nanovaccine. It has been reported that molecules of 20–200 nm can efficiently enter the lymphatic system, with an optimal size of around 30–40 nm [56, 57]. The small size of nanoparticles promoted the draining of nanovaccine to lymph nodes, which was evidenced by the in vivo accumulation of LDC@ZnP NPs in TDLNs. After NIR irradiation, the nanovaccine showed good photothermal safety to BMDCs, indicating the non-toxic to immune cells. It also displayed better in vitro, intracellular and in vivo photothermal effects than free melanin, which could be a result of aggregation of melanin by nanoparticle encapsulation [58]. When incubating with DCs, the nanovaccine can simultaneously promote the proliferation and maturation of BMDCs, demonstrating by the increased cell proliferation, upregulated CD11c^+^CD86^+^ cells and increased secretion of IL-6 and TNF-α. The immune stimulation to DCs could be resulted from the self-adjuvant effect of ZnP NPs [22]. Moreover, with the ability to promote the activation or maturation of innate immune cells, the mild heat generated by laser exposure further amplified the maturation of DCs [19].

Since the immunostimulatory and mild photothermal effect had been evidenced in vitro, the in vivo antitumor efficacy of DC hybrid nanovaccine was investigated in MC38 tumor-bearing mice. It was found that the nanovaccine showed pronounced tumor suppression by inhibiting the tumor growth and neovascularization but facilitating tumor necrosis. To understand the mechanism underlying the antitumor effect, the immune cells and factors in TDLNs, spleen, tumor microenvironment or serum were analyzed. To initiate the antitumor immunity, immature DCs need to uptake the tumor associated antigen and undergo maturation for antigen processing. After relocating to lymphoid organs, the primed DCs present the antigens to CD4^+^ and CD8^+^T cells to induce subsequent immune responses [59, 60]. With accumulation in TDLNs, the DC maturation of LDC@ZnP NPs treated groups was both increased in TDLNs, spleen and tumor. In the published research, Adpgk-loaded nanodiscs could elicit 47-fold greater frequencies of peptide-specific CTLs, indicating the induced antigen-specific antitumor T cell responses by Adpgk-loaded nanovaccine [8]. Moreover, in our previous work, HGP100 or TRP2 (melanoma associated antigenic peptide) was used as the model antigenic peptide to be loaded in L@ZnP NPs to study the antigen-specific T cell response. The results showed that peptide loaded L@ZnP NPs remarkably promoted the secretion of antigen-specific IFN-γ by T cells [22]. Here the activation and proliferation of CD8^+^ CTLs and CD4^+^ helper T cells were enhanced in Adpgk loaded L@ZnP NPs treated group, indicating L@ZnP-A NPs could induced antigen-specific immune responses. The antitumor immune responses were further improved when combined with mild heat. As the most important immune killer cells, cytotoxic CD8^+^ T lymphocytes need to cross the traffic vascular barriers and traffic into tumor microenvironment [61]. IL-6, a pyrogenic cytokine secreted under mild heat generation, is critical for lymphocyte trafficking to tumors [21]. The elevated levels of IL-6 in serum and tumor sections were identified in LDC@ZnP-MA + NIR treated mice, resulting in a favorable infiltration of CTLs in tumor. With increased maturation of DCs, proliferation and infiltration of CD8^+^ T cells and levels of immunostimulatory cytokines, the combined immunotherapy mediated by antigen peptide and mild heat achieved boosted antitumor immune responses.

## Conclusion

In summary, we successfully prepared a kind of DC hybrid nanovaccine coencapsulating Adpgk and melanin for combined immunotherapy against MC38. The nanovaccine can actively target to homologous DCs with enhanced cellular uptake in vitro and in vivo. When irradiated by 808 nm laser, the nanovaccine generated comparable heat for mild phototherapy. After in vivo administration, the nanovaccine can effectively drain to tumor draining lymph nodes as well as penetrate in tumor. The nanovaccine showed satisfied antitumor effect with the peptide and mild heat-inspired combined immunotherapy. The boosting DC maturation, CD8^+^ and CD4^+^ T cell proliferation and infiltration as well as immunostimulatory cytokines secretion contributed to the antitumor efficacy of nanovaccine. Therefore, this kind of DC hybrid nanovaccine for combinational immunotherapy provided a promising strategy in cancer treatment.

## Methods

### Materials and reagents

Zinc nitrate (Zn(NO_3_)_2_·6H_2_O, AR), disodium hydrogen phosphate (Na_2_HPO_4_, AR) and cyclohexane were purchased from Sinopharm Chemical Reagent Co., Ltd. (China).

Igepal CO-520 and lipopolysaccharide (LPS) were purchased from Sigma-Aldrich. Dioleoyl phosphatidic acid (DOPA) was purchased from Avanti Polar Lipids, Inc. (USA). 2-dioleoyl-snglycero-3-phosphocholine (DOPC), 1,2-dioleoyl-sn-glycero-3-phosphoethanolami-ne-N-[methoxy(polyethylene glycol)-2000] (DSPE-PEG2000) were purchased from Corden Pharma Switzerland LLC (Switzerland). Cholesterol were obtained from Lipoid GmbH (Germany). Adpgk peptide with hydrophilic modification (RRDDASMTNMELM) was synthesized by Bioyears (Wuhan Bioyeargene Biosciences Co. Ltd., China). RPMI 1640 medium was purchased from Thermo Fisher Scientific Inc. (USA). FBS was obtained from Procell Life Science & Technology (China). 1% penicillin/streptomycin, 1,1′-dioctadecyl-3,3,3′,3′-tetramethylindocarbocyanine perchlorate (DiI), 1,1′-dioctadecyl-3,3,3′,3′-tetramethylindotricarbocyanine iodide (DIR), and BCA protein assay kit were bought from Beyotime Biotechnology (China). 4′,6-diamidino-2-phenylindole (DAPI) was purchased from Aspen (South Africa). 3- [4, 5]-dimethylthiahiazo (-z-y1)-3,5-di- phenytetrazoliumromide (MTT) was purchased from Biofroxx (Germany). Granulocyte-macrophage colony stimulating factor (GM-CSF) and Interleukin 4 (IL-4) were purchased from PeproTech Inc. (USA). Anti-mouse monoclonal antibodies against CD11c, CD86, CD16/CD32, CD45, CD3e, CD4 and CD8a were purchased from BD Bioscience (USA). Enzyme-linked immunosorbent assay (ELISA) Kits for IL-6 and TNF-α were purchased from Dakewe Biotech Co., Ltd. (China). Spleen lymphocyte isolation kits were purified by TBD (China). Tumor infiltrating lymphocytes isolation kit was purchased from Beijing Solarbio Science & Technology Co., ltd. (China).

### Cell line and animals

Murine MC38 cells were cultured in RIPM 1640 medium supplemented with 10% fetal bovine serum (FBS) and 1% penicillin-streptomycin solution in a humidified atmosphere incubator with 5% CO_2_ at 37 °C. Six to eight weeks old female C57BL/6 mice were purchased from Hubei Provincial Center for Disease Prevention and Control, China. All mice were fed under specific pathogen-free (SPF) condition and treated according to the regulations of Laboratory Animals Ethics of Huazhong University of Science and Technology (HUST). The study was approved ([2022] IACUC Number: 3074) by the Institutional Animal Care and Use Committee at Tongji Medical College, HUST.

### Preparation of peptide and melanin co-loaded nanoparticles

Adpgk was dissolved in Zn(NO_3_)_2_ solution (pH 8.4, 500 mM) and added into 5 mL cyclohexane/Igepal CO-520 (71:29). 0.1, 0.2, 0.3, 0.4 and 0.5 mg melanin (20 mg/mL dissolved in concentrated ammonia) as well as 62.5 µL DOPA (20 mg/mL) were added into Zn phase, respectively. Na_2_HPO_4_ solution (100 mM) and DOPA were dropped into cyclohexane/Igepal CO-520 to obtain P phase. After stirring for 30 min, P phase was added to Zn phase dropwise and continued to stir for 2 h. Then an equal volume of absolute ethanol was added for demulsification. The mixture was centrifuged at 11,000 rpm for 15 min and washed twice with absolute ethanol. The co-loaded zinc phosphate nanoparticles (ZnP NPs) were obtained and dispersed in chloroform. To further prepare lipid enveloped ZnP NPs (L@ZnP NPs), ZnP NPs in chloroform were mixed with DOPC, DSPE-PEG2000, and cholesterol (4:1:4), subjected into rotary evaporator (Shanghai Xiande Experimental Instrument Co. Ltd., China) to form a thin film and hydrated by PBS.

### Obtain of DC membrane protein

C57BL/6 mice were used to obtain bone marrow derived dendritic cells (BMDCs). The marrow cavities of femurs and tibias were rinsed by cold PBS to harvest the precursor cells. Then the cells were cultured in complete medium supplemented with GM-CSF (20 ng/mL) and IL-4 (5 ng/mL). BMDCs were obtained after culturing for 7 days. The membrane proteins of DCs were isolated by Cell Membrane Protein Extraction Kit according to manufacture instruction (Beyotime Biotechnology, China) and quantified by BCA protein assay kit.

### Preparation of DC hybrid ZnP NPs

Firstly, blank L@ZnP NPs were prepared. Instead of simple PBS, PBS containing different amounts of DC membrane proteins (protein/phospholipid weight ratios of 1:0, 1:100, 1:200, 1:300, 1:400) were used for hydration. After collection and freeze-drying, the DC hybrid nanoparticles (LDC@ZnP NPs) were detected by Differential Scanning Calorimeter (TA, USA) to optimize the protein amount. Similarly, Adpgk or melanin loaded or co-loaded LDC@ZnP NPs were prepared with the above method, and noted as LDC@ZnP-A NPs, LDC@ZnP-M NPs and LDC@ZnP-MA NPs, respectively.

### Detection of Adpgk and melanin

High performance liquid chromatography (HPLC) and microplate reader were used to detect Adpgk and melanin, respectively. To determine Adpgk, the NPs were solved in acetonitrile: H_2_O (V/V 1:1) containing 0.1% TFA. After filtering by 0.22 μm membrane, the samples were detected by HPLC (Agilent-1260 InfinityII, USA) at the wavelength of 220 nm. For melanin detection, the NPs were disrupted by ammonia water and detected by enzyme calibration (PerkinElmer, USA) at 475 nm.

### Characterization of LDC@ZnP NPs

LDC@ZnP NPs were suspended in pH 7.4 PBS and dropped on EM grids (Beijing Zhongjingkeyi Technology, China). The morphology was observed by transmission electron microscope (TEM, HITACHI-HT7800, Japan). The hydration particle size and zeta potential of LDC@ZnP NPs were measured by Nano-ZS dynamic light scattering (DLS, Malvern, UK). For stability study, LDC@ZnP NPs were dispersed in PBS and FBS, respectively. The size change in continuous 7 days was monitored by DLS.

### In vitro photothermal effect

LDC@ZnP-M NPs or equivalent free melanin (50 µg/mL) was exposed to an 808 nm laser at 1, 1.5 and 2 W/cm^2^. After irradiating for 1, 2, 3, 4, 5 min, the temperature was recorded by infrared thermal imaging camera (FLIR-E8-XT, USA), respectively. To study photothermal effect of LDC@ZnP-M nanoparticles with different concentrations, the nanoparticles were diluted into a series of concentrations with PBS and irradiated with the power optimized above, the real-time temperature was recorded by thermal imaging camera.

### The intracellular photothermal effect

BMDCs were plated on 24-well plates at 150, 000 cells per well and cultured overnight to obtain a semi-attached status. A pipette was used to carefully suck away the old medium but retain 10% to avoid cell loss. Another 90% fresh medium containing PBS, LDC@ZnP-M NPs and equivalent free melanin (50 µg/mL) was gently added to the culture plate along the wall of the hole. After incubating with BMDCs for 4 h, the medium was replaced with PBS. Then the cells were placed at room temperature and irradiated with an 808 nm laser. The real-time temperature in 5 min was recorded by infrared thermal imaging camera.

### Uptake of LDC@ZnP NPs by BMDCs

BMDCs were plated on 24-well plates at 150, 000 cells per well and cultured overnight. LZnP NPs and LDC@ZnP NPs were labeled with fluorescent dye DiI. Then the cells were incubated with the DiI labeled NPs for 1, 2, 4, and 6 h, respectively. The direct and quantitative uptake behaviors by BMDCs were detected by confocal laser scanning microscope (Leica, USA) and flow cytometer (Beckman coulter, USA).

### Evaluation of photothermal safety to DCs

BMDCs were inoculated into 96-well plates at 20, 000 cells per well and cultured overnight. LDC@ZnP NPs with different concentrations were added into the cell culture plates, respectively. After incubation with the cells for 4 h, cell supernatant was removed and replaced with fresh blank medium. Then the cells were placed on a thermostat controlled hot plate to maintain the cells at 37 ℃, irradiated with 808 nm laser from the bottom and continuously cultured for another 20 h. The survival rate of cells was detected by MTT assay.

### The immune stimulation of LDC@ZnP NPs to BMDCs

BMDCs were plated on 24-well plates at 150, 000 cells per well and cultured overnight. PBS with or without NIR (PBS +/- NIR), free melanin (10 µg/mL) and Adpgk (4 µg/mL) mixture with or without NIR (Free M + A +/- NIR), equivalent LDC@ZnP-M + NIR, LDC@ZnP-A, LDC@ZnP-MA + NIR and the positive control LPS (1 µg/mL) were added to the cells. The cells were incubated with different formulations for 4 h and replaced with fresh medium. For the groups with NIR irradiation, the cells were placed on the thermostat controlled hot plate (37 ℃) and exposed to 2 W/cm^2^ laser for 2 min. After another 20 h of culture, the cells were collected, stained with anti-mouse CD11c and anti-mouse CD86 antibodies and detected by flow cytometer. Meanwhile, IL-6 and TNF-α in the supernatant were detected by ELISA kit.

### The reflux of LDC@ZnP NPs to lymph nodes

DiI labeled LZnP NPs and LDC@ZnP NPs were administered subcutaneously to the armpit and femoral groin regions of C57BL/6 mice. After 24 h, the mice were sacrificed. The axillary lymph nodes (ALNs) and inguinal lymph nodes (ILNs) were collected and made for frozen slices. Cell nuclei were stained with DAPI. The reflux of NPs to lymph nodes was observed under confocal microscope.

### The in vivo uptake of LDC@ZnP NPs by DCs

DiI labeled LZnP and LDC@ZnP NPs were administered subcutaneously to the armpit and femoral groin regions of C57BL/6 mice. 12 and 24 h later, ALNs and ILNs were collected for isolating lymphocytes. The cells were stained with anti-mouse CD11c antibodies and detected by flow cytometer.

### The in vivo biodistribution of LDC@ZnP NPs

For in vivo biodistribution study, DIR labeled LZnP NPs and LDC@ZnP NPs were prepared similarly as DiI labeled NPs. C57BL/6 mice were injected subcutaneously with 2.5 × 10^5^ MC38 cells. When the tumor volume increased to approximately 500 mm^3^, the mice were randomly divided into three groups. Free DIR, LZnP NPs or LDC@ZnP NPs were injected subcutaneously to the right flank of mice. At scheduled time points (4, 8, 24, 32, 48 h), the mice were anesthetized by intraperitoneal injection of 0.3% sodium pentobarbital and monitored by live imager (Bruker, Germany). At 48 h post injection, the mice were sacrificed and tissues including tumor, TDLNs, heart, liver, spleen, lung and kidney were harvested for *ex viv*o imaging.

###  The in vivo photothermal effect

C57BL/6 mice were injected subcutaneously with 2.5 × 10^5^ MC38 cells. PBS, Free melanin (50 µg) equivalent LDC@ZnP-M NPs or LDC@ZnP-MA NPs were injected to the tumor-bearing mice. After injecting for 24 h, the mice were anesthetized by intraperitoneal injection of 0.3% sodium pentobarbital. The tumor was irradiated with an 808 nm laser at 2 W/cm^2^ for 3 min. The temperature was recorded by thermal imaging camera.

### The in vivo antitumor effect of LDC@ZnP NPs

C57BL/6 mice were injected subcutaneously with 1.25 × 10^5^ MC38 cells. Two days after tumor inoculation, the mice were treated with PBS, PBS + NIR, Free M + A (50 µg melanin, 20 µg Adpgk), Free M + A + NIR, LDC@ZnP-M NPs + NIR, LDC@ZnP-A NPs and LDC@ZnP-MA NPs + NIR for three times, respectively. The NIR irradiation was conducted at 24 h post administration. During the treatment, the mental and physiological conditions of the mice were observed, and the tumor volume was recorded. For mental status observing, the responses of mice to the aversion condition of being held and hung upside down were monitored. On day 20, the mice were sacrificed. Tumor was harvested, weighed, and calculated for tumor inhibition rate. Hematoxylin-eosin (HE) was used to stain the tumor, and the apoptotic necrosis inside the tumor was observed by the fluorescence microscope (IX73, OLYMPUS, Japan). CD31 immunofluorescent analysis of tumor tissues was performed to observe the angiogenesis.

### Mechanism study of nano-vaccine mediated antitumor effect

On the third day after the last immunization, TDLNs, spleen, and tumors were collected. The lymphocytes were purified and stained with anti-mouse CD 11c, anti-mouse CD86, anti-mouse CD16/CD32, anti-mouse CD45, anti-mouse CD3e, anti-mouse CD4 and anti-mouse CD8a, respectively. The maturation and proliferation of DCs, CD4^+^ and CD8^+^ T cells were detected by flow cytometer. Furthermore, the serum levels of IL-6 and TNF-α were measured using ELISA.

### Statistical analysis

Data were shown as mean ± standard deviation (SD). Data were processed by Prism v8 (GraphPad, USA) and analyzed by One-way analysis of variance (ANOVA) or Student’s t test. *p < 0.05 indicated that there were significant statistical differences.

### Supplementary Information

**Additional file 1: Figure S1.** TEM image of blank ZnP NPs dispersed in chloroform, scale bar = 100 nm. **Figure S2.** SDS-PAGE of DC (1), extracted membrane protein (2) and LDC@ZnP NPs (3). **Figure S3.** Photothermal profiles of LDC@ZnP-MA NPs (50 μg/mL melanin) under 2 W/cm^2^ irradiation. **Figure S4.** Photothermal safety of LDC@ZnP-M NPs under the irradiation of 2 W/cm2 for 3 min (n = 4). **Figure S5.** The quantitative analysis of mean fluorescence intensity in ALNs and ILNs (n = 3), *p < 0.05. **Figure S6.** **A** Ex vivo images of heart, liver, spleen, lung and kidney at 48 h post-injection. **B** Fluorescence quantitation of free DIR, L@ZnP NPs and LDC@ZnP NPs in ex vivo tissues (n = 4), ns: not significant. **Figure S7.** The in vivo photothermal images (**A**) and temperature (**B**) of LDC@ZnP-MA NPs after irradiating with 808 nm laser for 2 min (n = 3), **p < 0.01. **Figure S8.** **A** The body of weight of mice with the treatment of PBS, PBS+NIR, Free M + A, Free M + A + NIR, LDC@ZnP-M NPs + NIR, LDC@ZnP-A NPs, LDC@ZnP-MA NPs + NIR, respectively (n =7). **B** Tumor inhibitory rate by contrast with PBS (n = 7), *p < 0.05, **p < 0.01. **Figure S9.** DC maturation in the spleen of PBS, PBS+NIR, Free M+A, Free M + A + NIR, LDC@ZnP-M NPs + NIR, LDC@ZnP-A NPs and LDC@ZnP-MA NPs + NIR treated mice (n = 4), *p < 0.05. **Figure S10.** The infiltration of DCs in tumor environment after different treatments (n = 4), *p < 0.05. **Figure S11.** CD4^+^ T cells in the TDLNs of PBS, PBS+NIR, Free M + A, Free M + A + NIR, LDC@ZnP-M NPs + NIR, LDC@ZnP-A NPs and LDC@ZnP-MA NPs + NIR treated mice (n = 4), *p < 0.05. **Figure S12.** CD4 ^+^ T cells in the spleen of PBS, PBS+NIR, Free M + A, Free M + A + NIR, LDC@ZnP-M NPs + NIR, LDC@ZnP-A NPs and LDC@ZnP-MA NPs+NIR treated mice (n = 4), *p < 0.05.

## Data Availability

Data will be made available on request.
