# Correction: Dendritic cell hybrid nanovaccine for mild heat inspired cancer immunotherapy

**DOI:** 10.1186/s12951-023-02140-6

**Published:** 2023-11-21

**Authors:** Chen Shi, Chen Jian, Lulu Wang, Chen Gao, Ting Yang, Zhiwen Fu, Tingting Wu

**Affiliations:** 1 Department of Pharmacy, Union Hospital, Tongji Medical College, Huazhong University of Science and Technology, Wuhan, 430022 China; 2 Hubei Province Clinical Research Center for Precision Medicine for Critical Illness, Wuhan, 430022 China; 3 Affiliated Hospital of Yunnan University, Kunming, 650000 China

**Correction: Journal of Nanobiotechnology (2023) 21:347**

10.1186/s12951-023-02106-8

Following publication of the original article [1], the figure legends in additional file were mistakenly marked in blue text. The correct version of ESM has been included in this correction and the original article [1] has been corrected.

### Electronic supplementary material

Below is the link to the electronic supplementary material.

Supplementary Material 1
