# Supplementary material for: Dendritic cell hybrid nanovaccine for mild heat inspired cancer immunotherapy
Source: J Nanobiotechnology. 2023 Sep 26;21:347. doi: 10.1186/s12951-023-02106-8 (PMC10521411; doi:10.1186/s12951-023-02106-8)
Supplement: Supplementary file 1 — Additional file 1: Figure S1. TEM image of blank ZnP NPs dispersed in chloroform, scale bar = 100 nm. Figure S2. SDS-PAGE of DC (1), extracted membrane protein (2) and LDC@ZnP NPs (3). Figure S3. Photothermal profiles of LDC@ZnP-MA NPs (50 μg/mL melanin) under 2 W/cm2 irradiation. Figure S4. Photothermal safety of LDC@ZnP-M NPs under the irradiation of 2 W/cm2 for 3 min (n = 4). Figure S5. The quantitative analysis of mean fluorescence intensity in ALNs and ILNs (n = 3), *p < 0.05. Figure S6. A Ex vivo images of heart, liver, spleen, lung and kidney at 48 h post-injection. B Fluorescence quantitation of free DIR, L@ZnP NPs and LDC@ZnP NPs in ex vivo tissues (n = 4), ns: not significant. Figure S7. The in vivo photothermal images (A) and temperature (B) of LDC@ZnP-MA NPs after irradiating with 808 nm laser for 2 min (n = 3), **p < 0.01. Figure S8. A The body of weight of mice with the treatment of PBS, PBS+NIR, Free M + A, Free M + A + NIR, LDC@ZnP-M NPs + NIR, LDC@ZnP-A NPs, LDC@ZnP-MA NPs + NIR, respectively (n =7). B Tumor inhibitory rate by contrast with PBS (n = 7), *p < 0.05, **p < 0.01. Figure S9. DC maturation in the spleen of PBS, PBS+NIR, Free M+A, Free M + A + NIR, LDC@ZnP-M NPs + NIR, LDC@ZnP-A NPs and LDC@ZnP-MA NPs + NIR treated mice (n = 4), *p < 0.05. Figure S10. The infiltration of DCs in tumor environment after different treatments (n = 4), *p < 0.05. Figure S11. CD4+ T cells in the TDLNs of PBS, PBS+NIR, Free M + A, Free M + A + NIR, LDC@ZnP-M NPs + NIR, LDC@ZnP-A NPs and LDC@ZnP-MA NPs + NIR treated mice (n = 4), *p < 0.05. Figure S12. CD4 + T cells in the spleen of PBS, PBS+NIR, Free M + A, Free M + A + NIR, LDC@ZnP-M NPs + NIR, LDC@ZnP-A NPs and LDC@ZnP-MA NPs+NIR treated mice (n = 4), *p < 0.05. [file 12951_2023_2106_MOESM1_ESM.docx]

**Additional file information**

**Dendritic cell hybrid nanovaccine for mild heat inspired cancer immunotherapy**

Chen Shi^1, 2^, Chen Jian^1^, Lulu Wang^1^, Chen Gao^1^, Ting Yang^3^, Zhiwen Fu^1, 2^,

Tingting Wu^1, 2^*

^1^ Department of Pharmacy, Union Hospital, Tongji Medical College, Huazhong University of Science and Technology, Wuhan, 430022, China.

^2^ Hubei Province Clinical Research Center for Precision Medicine for Critical Illness, Wuhan, 430022, China.

^3^ Affiliated Hospital of Yunnan University, Kunming, 650000, China.

*Corresponding author. E-mail: tingtingwu1202@163.com


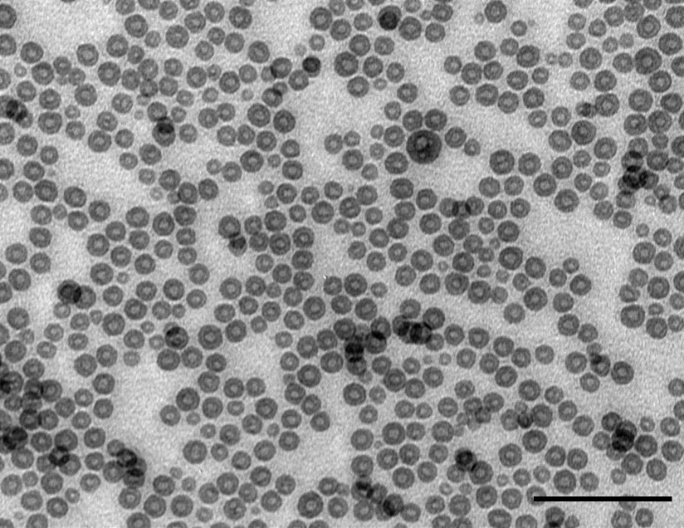


**Figure S1.** TEM image of blank ZnP NPs dispersed in chloroform, scale bar = 100 nm.


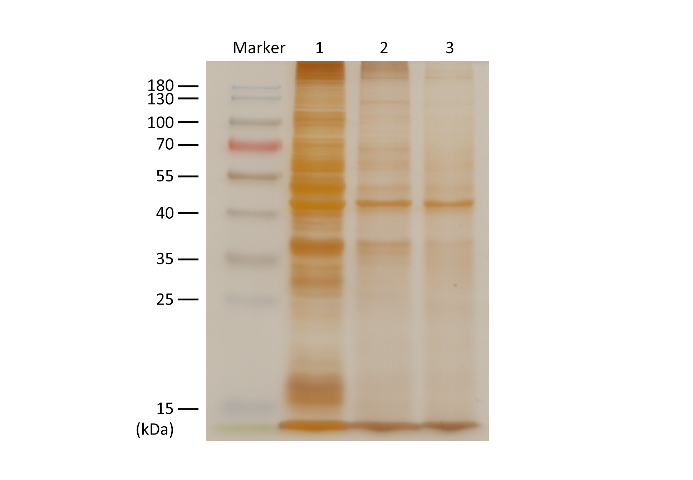


**Figure S2.** SDS-PAGE of DC (1), extracted membrane protein (2) and LDC@ZnP NPs (3).


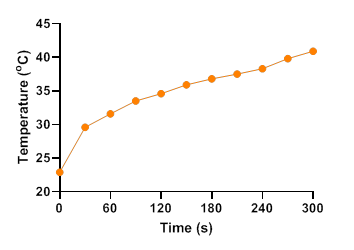


**Figure S3.** Photothermal profiles of LDC@ZnP-MA NPs (50 μg/mL melanin) under 2 W/cm^2^ irradiation.


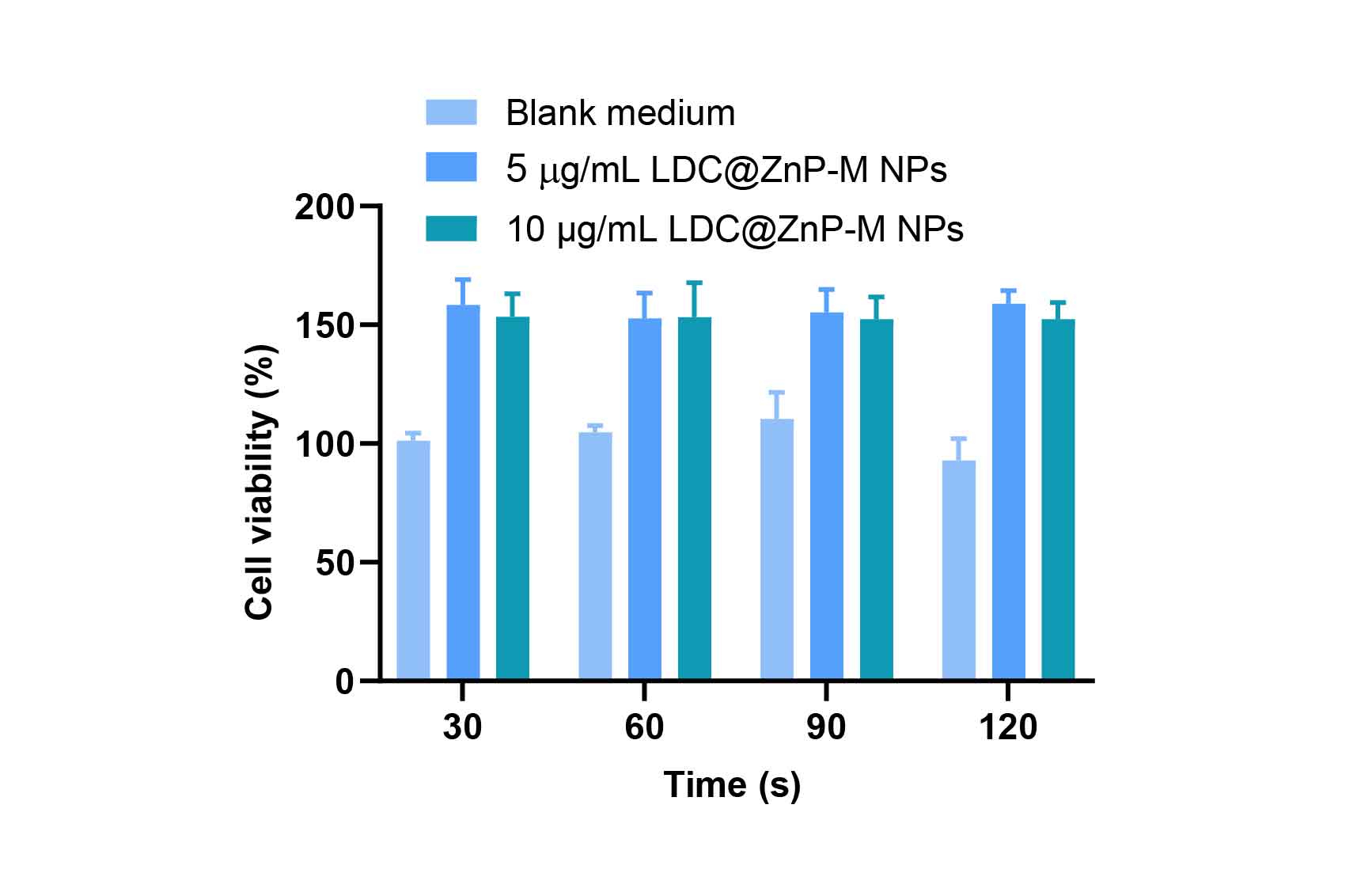


**Figure S4.** Photothermal safety of LDC@ZnP-M NPs under the irradiation of 2 W/cm^2^ for 3 min (n = 4).

**Figure S5.** The quantitative analysis of mean fluorescence intensity in ALNs and ILNs (n = 3), **p* < 0.05.


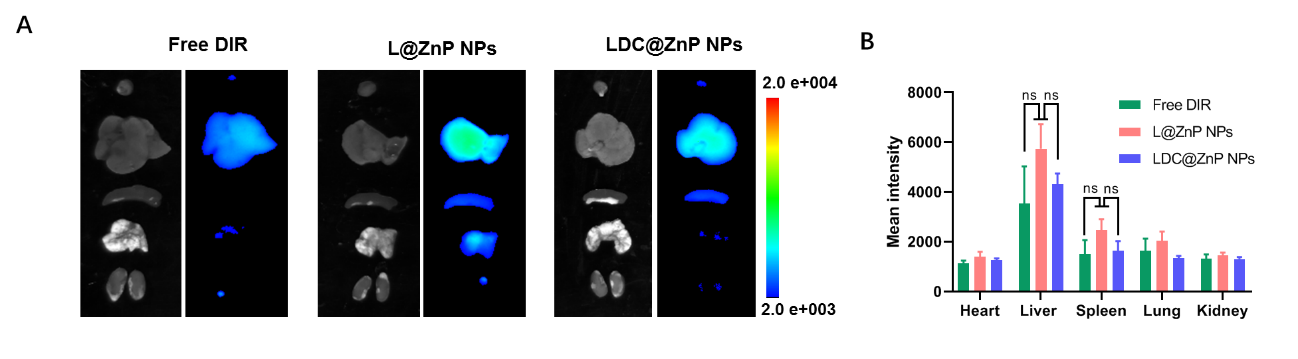


**Figure S6.** (A) *Ex vivo* images of heart, liver, spleen, lung and kidney at 48 h post-injection. (B) Fluorescence quantitation of free DIR, L@ZnP NPs and LDC@ZnP NPs in *ex vivo* tissues (n = 4), ns: not significant.


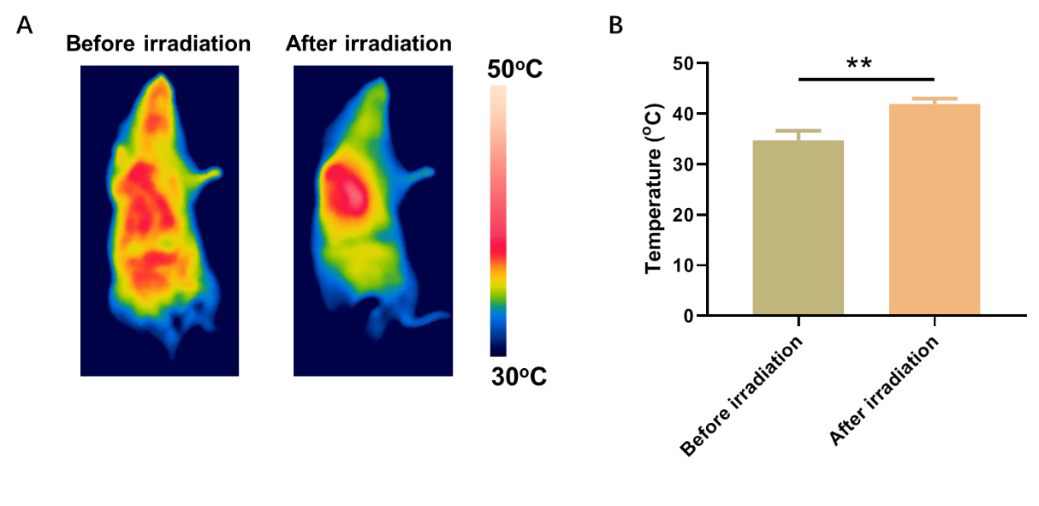


**Figure S7.** The *in vivo* photothermal images (A) and temperature (B) of LDC@ZnP-MA NPs after irradiating with 808 nm laser for 2 min (n = 3), ***p* < 0.01.


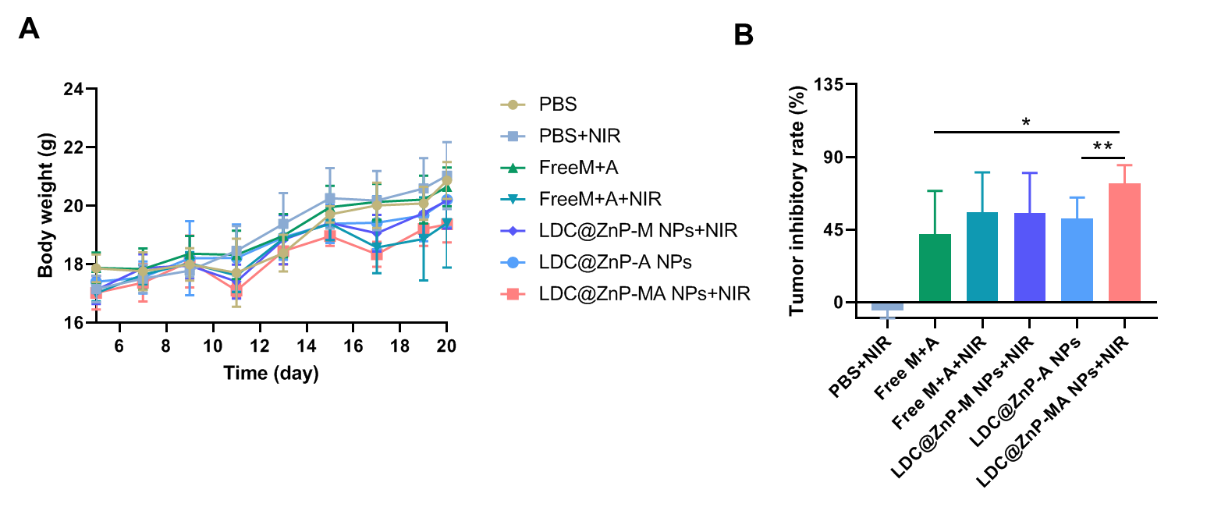


**Figure S8.** (A) The body of weight of mice with the treatment of PBS, PBS+NIR, Free M+A, Free M+A+NIR, LDC@ZnP-M NPs+NIR, LDC@ZnP-A NPs, LDC@ZnP-MA NPs+NIR, respectively (n =7). (B) Tumor inhibitory rate by contrast with PBS (n = 7), **p* < 0.05, ***p* < 0.01.


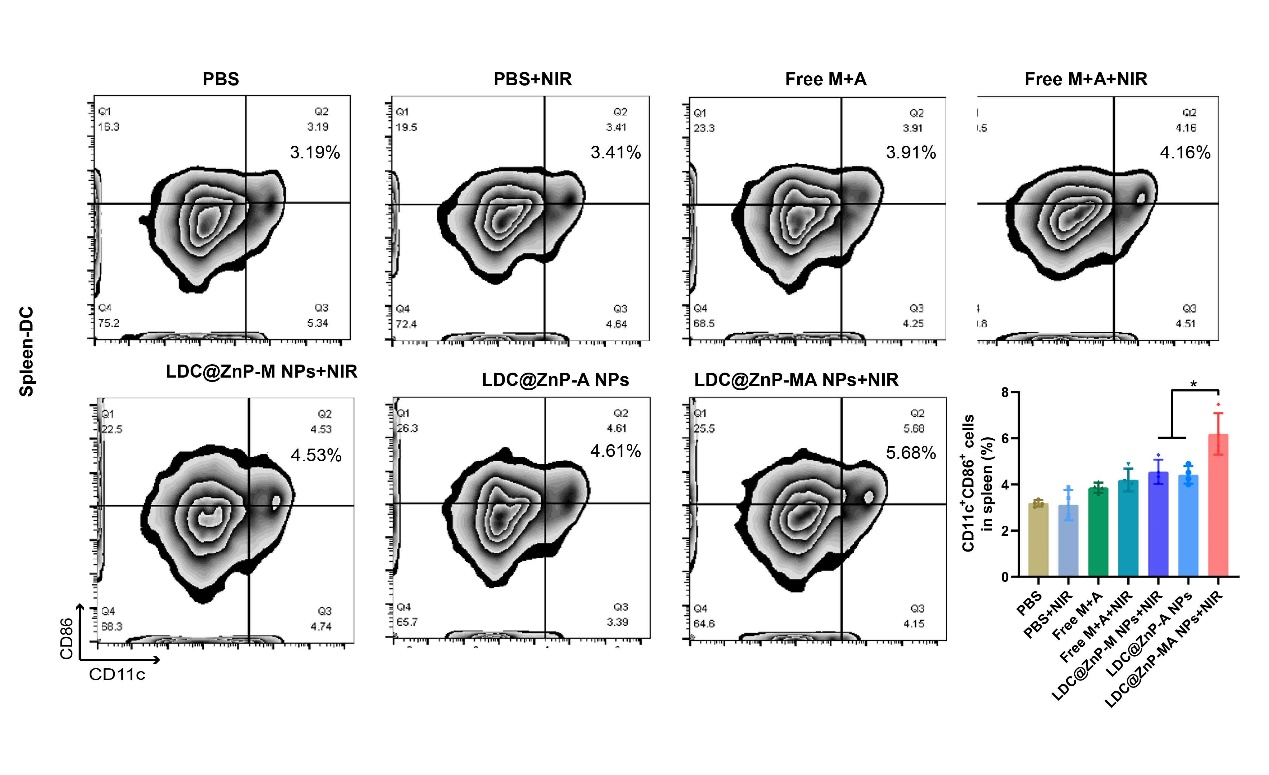


**Figure S9.** DC maturation in the spleen of PBS, PBS+NIR, Free M+A, Free M+A+NIR, LDC@ZnP-M NPs+NIR, LDC@ZnP-A NPs and LDC@ZnP-MA NPs+NIR treated mice (n = 4), **p* < 0.05.


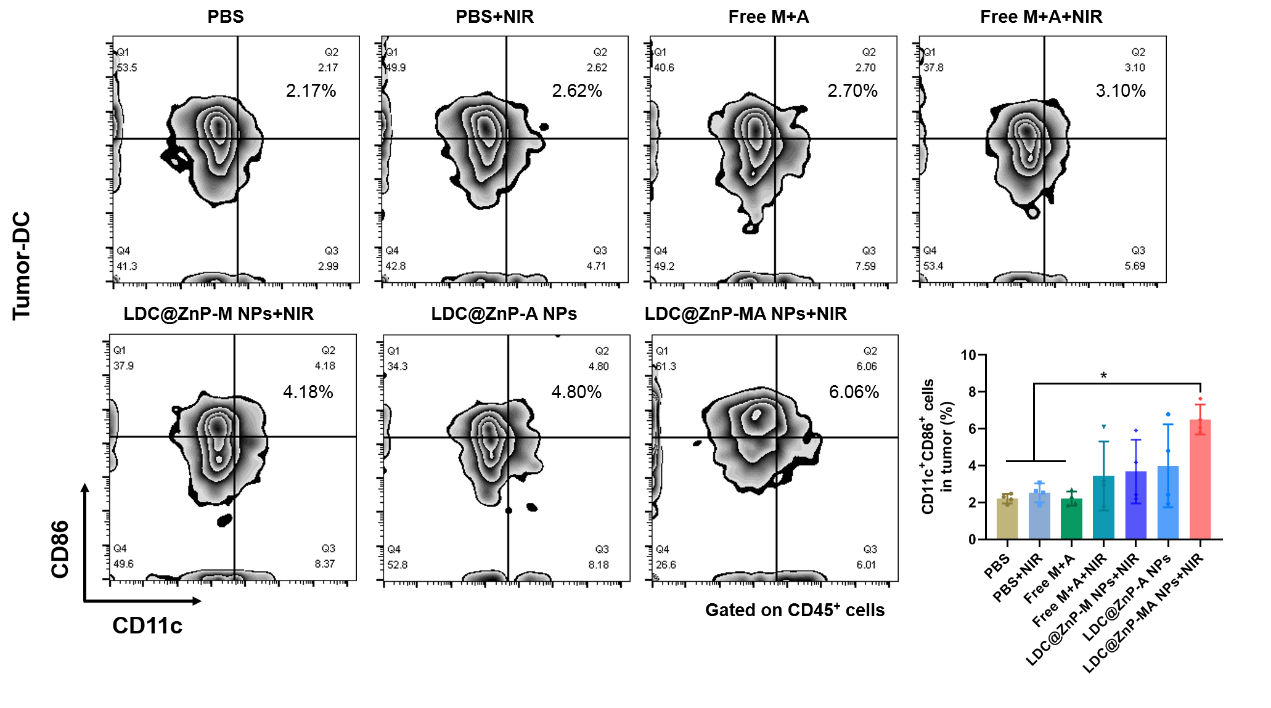


**Figure S10.** The infiltration of DCs in tumor environment after different treatments (n = 4), **p* < 0.05.

.


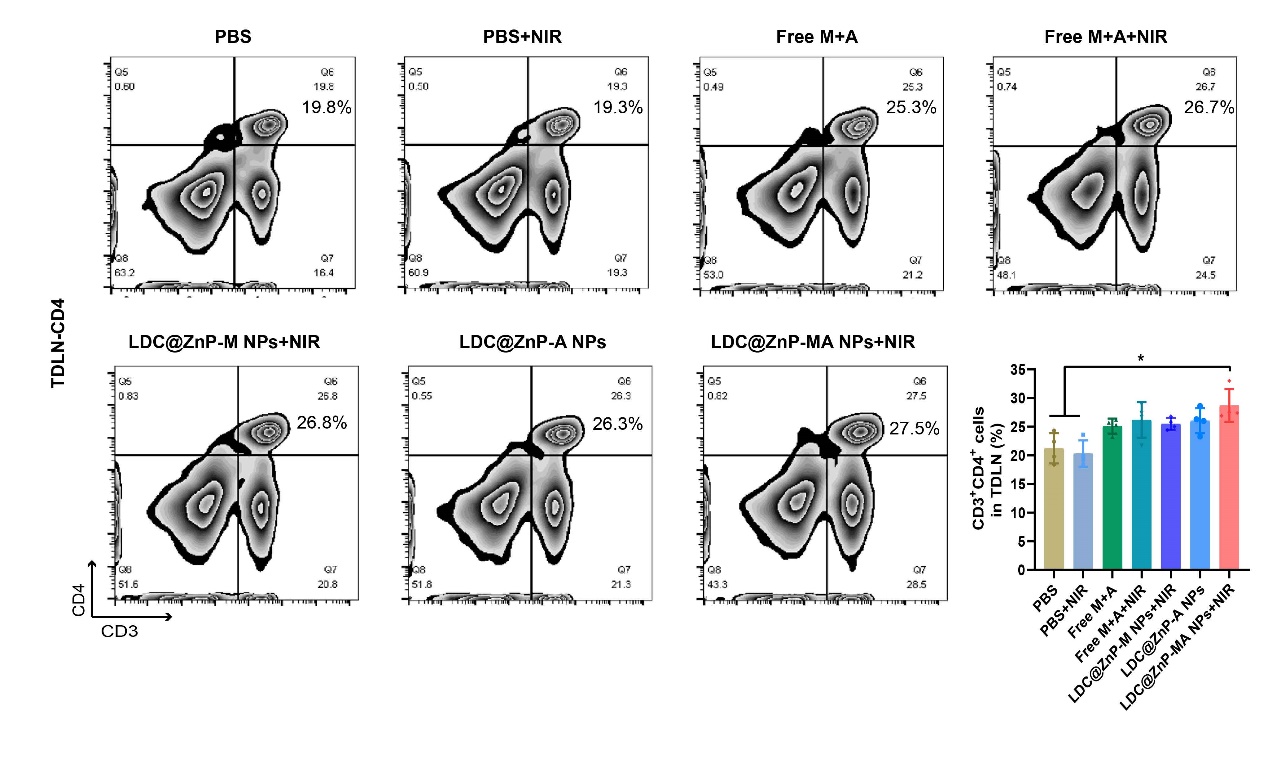


**Figure S11.** CD4^+^ T cells in the TDLNs of PBS, PBS+NIR, Free M+A, Free M+A+NIR, LDC@ZnP-M NPs+NIR, LDC@ZnP-A NPs and LDC@ZnP-MA NPs+NIR treated mice (n = 4), **p* < 0.05.


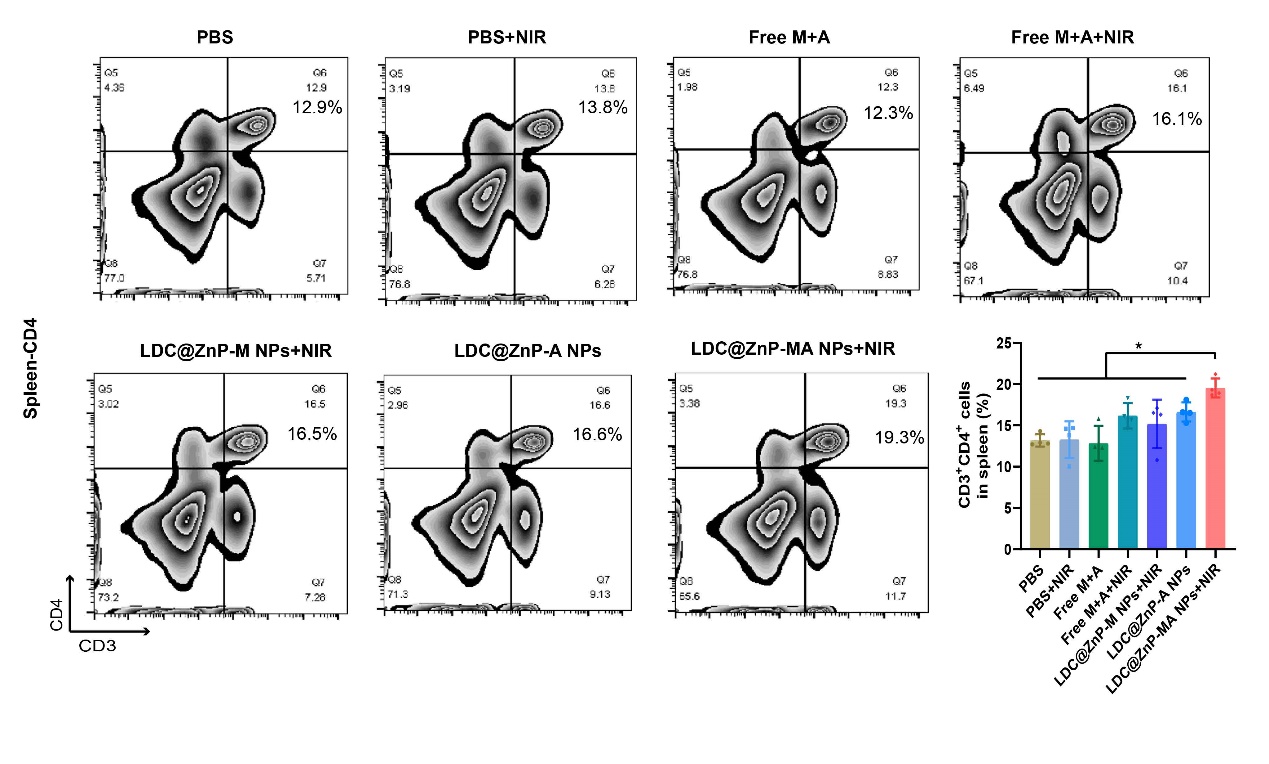


**Figure S12.** CD4^+^ T cells in the spleen of PBS, PBS+NIR, Free M+A, Free M+A+NIR, LDC@ZnP-M NPs+NIR, LDC@ZnP-A NPs and LDC@ZnP-MA NPs+NIR treated mice (n = 4), **p* < 0.05.
